# The bulb retouchers in the Levant: New insights into Middle Palaeolithic retouching techniques and mobile tool-kit composition

**DOI:** 10.1371/journal.pone.0218859

**Published:** 2019-07-05

**Authors:** Laura Centi, Iris Groman-Yaroslavski, Neta Friedman, Maya Oron, Marion Prévost, Yossi Zaidner

**Affiliations:** 1 Department of Prehistoric Archaeology, Institute of Archaeology, The Hebrew University of Jerusalem, Jerusalem, Israel; 2 Use-Wear Analysis Laboratory, Zinman Institute of Archaeology, University of Haifa, Haifa, Israel; 3 Department of Prehistory, Israel Antiquities Authority, Jerusalem, Israel; Universita degli Studi di Ferrara, ITALY

## Abstract

In this paper we describe two assemblages of flint retouchers or “bulb retouchers” retrieved from Nesher Ramla and Quneitra, two Middle Palaeolithic, open-air sites in the Levant. The site of Nesher Ramla yielded the largest assemblage of bulb retouchers (n = 159) currently known, allowing a detailed investigation of this poorly known phenomenon. An extensive experimental program and use-wear analysis enabled us to characterize the different sets of traces related to the retouching activity and to identify different motions applied by the knappers in the course of this action. In both sites, blanks used as bulb retouchers were almost exclusively retouched items, with a special emphasis on convergent morphotypes in Nesher Ramla. The use of retouched items as bulb retouchers is a common trait over different time spans and geographical areas. Our data suggests that bulb retouchers were versatile, multi-purpose tools with a long use-life, transported over long distances as components of the hunter-gatherer mobile tool kit. The high frequencies of bulb retouchers within some archaeological units of Nesher Ramla appear to be connected to the highly curated nature of the lithic assemblages, in turn reflecting a high mobility of the human groups that produced them.

## Introduction

The modification of the shape of lithic blanks through retouch is an important technological aspect of the behaviour of past populations, documented since the Lower Palaeolithic. By retouch, humans shaped stone flakes into tools of recurrent morphologies, many of which are used by archaeologists as markers of different cultural entities and phases of cultural evolution. Besides determining the final shape of a tool, the retouch of a blank edge might improve its sturdiness and create a profile suited to different kinds of activities. It can also be intended to re-sharpen a worn edge, extending the blank’s use-life.

Retouch was executed using different types of tools known as retouchers. The earliest retouched tool forms do not exhibit invasive, finely-executed retouch and thus probably did not require use of specific retouchers. Artefacts identified as retouchers begin to occur in the archaeological record during the late Lower Palaeolithic [1–7] when retouched tools became more standardized and the retouch more regular. During the Middle Palaeolithic period retouchers made of both organic and inorganic materials became a common component of the hunter-gatherers tool kit [1,8–11]. The best-known type of organic retouchers are bone retouchers (made of bone, tooth or antler), which have been identified in numerous Late Lower and Middle Palaeolithic sites [1–23]. Bone retouchers are generally considered as low-mobility expedient tools, in most cases selected from pieces of food waste and abandoned after a short use-life [9,23].

The best-known type of inorganic retouchers are pebbles, documented at several sites in Western Europe [4,24–27] and Crimea [14,19,20,28]. The use of pebble retouchers is considered to be a planned behaviour, where humans selected pebbles and transported them into the sites according to their shape (often flat and elongated), density and specific ranges of weight. Contrary to what is usually suggested for bone retouchers, pebble retouchers are thought to have been used over prolonged periods of time, as suggested by the intensity and superimposition of marks on their surfaces [23]. This is likely, due to their greater resistance to impact and the greater number of possible working surfaces compared to bone retouchers [20]. In addition, during the Lower and Middle Palaeolithic, cores and handaxes were occasionally used as retouchers [29,30].

A third, rarely mentioned type of inorganic retoucher are the “bulb retouchers” [31]. These are chipped stone artefacts that exhibit a concentration of marks, such as pits and incipient cones on the bulb area of the ventral face. The convex part of the bulb of percussion was used to strike the edges of other stone blanks in order to modify their original shape. This function was reconstructed based on the similarities between the marks observed on bulb retouchers and on other types of retouchers, as well as on few experimental studies [31–33].

**Fig 1:**
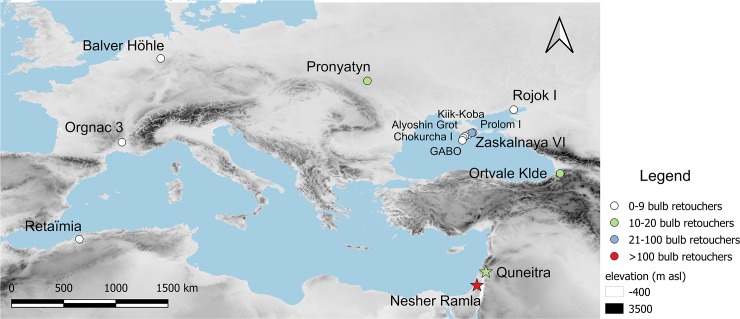
Location of the two studied sites and distribution of other bulb retouchers assemblages. The colours indicate the size of the assemblages. Nesher Ramla and Quneitra, the current case-studies, are indicated with stars. Map created with *QGIS*, based on digital elevation model freely distributed by GTOPO 30, USGS.

The bulb retouchers were first recognized by Praslov [34] in the Crimean site of Rojok I (Fig 1). Since then, bulb retouchers have been reported in several other sites in the Crimean Peninsula: Zaskalnaya VI [14], La Gouba [35], Alyoshin Grot [36], Chokurcha I [21], Prolom I [28], Kiik-Koba [28]. They are also known outside Crimea from a handful of sites: Pronyatyn in Ukraine [37], Ortvale Klde in Georgia [31] Retaïmia Cave in Algeria [32], Orgnac 3 in France [33] and Balver Höhle in Germany (Jöris, pers comm). In each of these assemblages, bulb retouchers occur in small numbers (1–10 items). It has been suggested that these were ad-hoc tools, expediently used over short periods of time [31], and that blanks were selected mainly according to thickness and bulbar mass. To date, bulb retouchers have been retrieved only from Middle Palaeolithic contexts.

Perhaps due to their small numbers across sites, bulb retouchers remain a poorly investigated phenomenon. Here we present two assemblages of bulb retouchers from the Levantine Middle Palaeolithic open-air sites of Nesher Ramla [38] and Quneitra [39]. The focus of this work is the bulb retoucher assemblage of Nesher Ramla, which originates from four stratigraphic units and is the largest known to date from a single site (n = 159). The unique size of this assemblage allowed the study of the bulb retouchers from a multidisciplinary perspective, with a better understanding of their general characteristics and what distinguish them from other types of retouchers. Quneitra’s bulb retouchers assemblage, is more similar in terms of its size (n = 13), to the ones previously reported from other sites. We report on Quneitra’s collection as an example of a more typical bulb retouchers’ assemblage, highlighting common traits and differences with the Nesher Ramla assemblage. The bulb retouchers were subjected to technological, morphological and micro-wear analysis, accompanied by an experimental program. The results of this study shed new light on how the bulb retouchers were used, on their role in the toolkit of the Middle Palaeolithic hominins and on their importance for the reconstruction of human mobility patterns and site function.

## The sites

### Nesher Ramla

**Fig 2:**
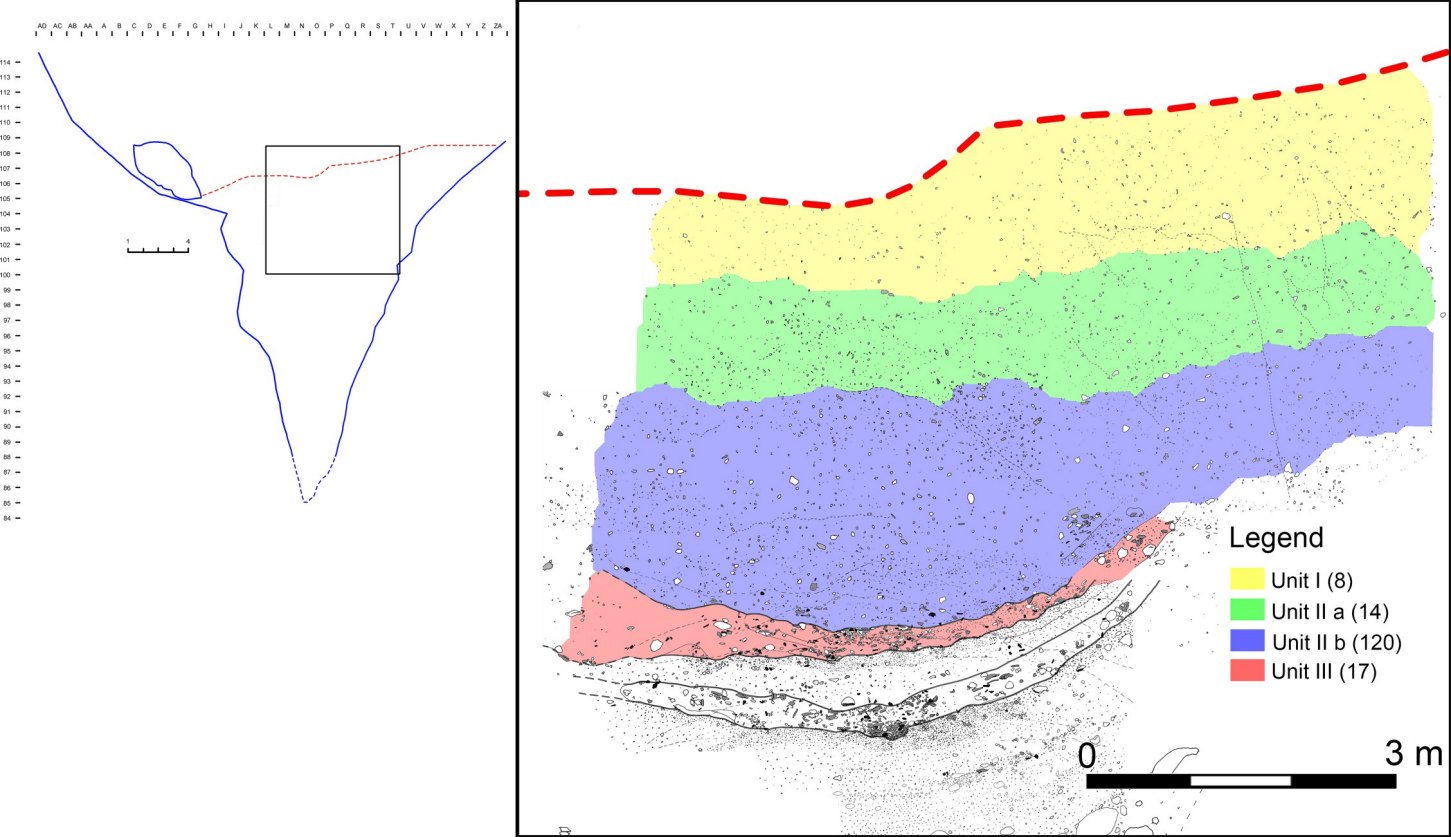
**Nesher Ramla karst depression profile (on the left) and stratigraphy (on the right).** The archaeological units object of this study are highlighted in different colours; the numbers in the brackets indicate the amount of bulb retouchers retrieved from each unit.

Nesher Ramla is an open-air site located on the western slopes of the Judean Hills, in central Israel (Fig 1), at 120–86 meters above sea level. The site was discovered during quarrying activities and excavated in a salvage operation directed by one of us (YZ) in 2010-2011(Israel Antiquities Authority Permits B355/2010, B368/2011) [38,40]. The archaeological material deposited *in situ* in a karst sinkhole formed within Senonian chalk of the ‘En Zetim Formation [40,41]. The sinkhole is funnel-shaped and is 34 m deep, with an eight meter thick archaeological sequence overlying 14 m of sterile sediment at the bottom and sealed by 12 m of sterile infill at the top (Fig 2). A series of OSL age estimates puts the human occupations at the site at the end of MIS 6 and during MIS 5, thus placing the entire sequence within the Middle Palaeolithic period [38]. The archaeological sequence was divided into six main units (I-VI) with some internal subdivision. The sequence shows dramatic changes in artefact densities, possibly reflecting different intensity of occupation of the site through time, with units III and V being the densest [40]. Human activities at the site were carried out *in situ* as testified by several fireplaces and ash lenses identified in units III and V, animal bones in anatomical articulation throughout the sequence along with lithic artefacts refitting and concentration of archaeological artefacts.

The lithic assemblage of Nesher Ramla comprises some 81,000 artefacts > 2 cm. The industry is characterized by the use of the Levallois method for the production of flakes, with Levallois blades and points as a minor component. An important element of Nesher Ramla lithic assemblage are the naturally backed knives (NBK's), which are numerous throughout the sequence and were the end-product of a separate reduction sequence [42]. When possible, lithic artefacts from Nesher Ramla were matched to surveyed flint sources at a radius of 15 km around the site, based on visual features such as flint colour and texture, following the methods of Ekshtain [43,44]. Flint types that did not match any of the surveyed sources were considered as non-local.

Retouched tools occur in relatively high frequencies in the assemblages, with side-scrapers and tools with lateral *tranchet* blow being the dominant types [42,45]. The modified items consist also of retouched flakes and Mousterian points (especially abundant in Unit IIB), while notches, denticulates and Upper Palaeolithic types are virtually absent. The maintenance of similar techno-typological traits along the entire archaeological sequence, including idiosyncratic features of Nesher Ramla industry (i.e. the high amount of NBK’s and the lateral *tranchet* blow technique), suggests cultural continuity in the human occupations of the site.

### Quneitra

**Fig 3:**
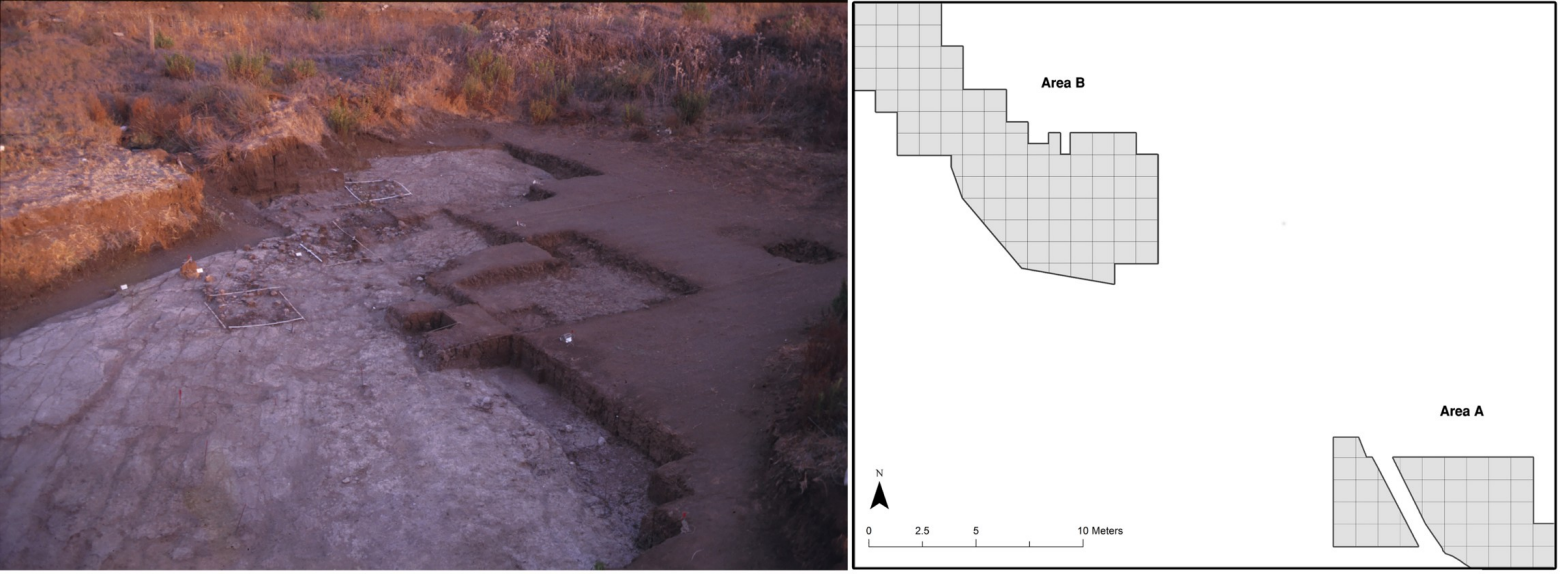
Quneitra Area B of excavation and site plan. On the left a picture showing Area B during excavation. On the right a plan of the two excavation areas (Area A and Area B).

Quneitra is a Middle Palaeolithic open-air site located on the northern Golan Heights (Fig 1). It was excavated 1982–1985 by a team of the Hebrew University of Jerusalem, directed by N. Goren-Inbar [39]. The excavation was conducted in two areas, Area A and Area B (Fig 3), in which a single extensive archaeological horizon (layer C) was identified. The horizon was 12–17 cm thick and yielded a large lithic (n = 12,598) and bone (n = 3,383) assemblages. A set of ESR age estimates on bovid teeth places the human occupation at 53±5.9 [46]. In Area B a small water body (seasonal pond) was identified, around which human activities took place.

The site has been interpreted as a hunting and butchery location, in which knapping activities were also carried out ([47], but see [48] for a different view). The lithic assemblage is characterized by the presence of two distinct reduction sequences: one on basalt for the production of massive scrapers and another on flint reduced through the Levallois method. Respectively, raw material sources were basalt flows in the immediate vicinity of the site and flint exposures located some 9–10 km to the north of the site. Flint can thus be considered a non-local raw material at Quneitra [48]. The first stages of the flint reduction sequence were carried on elsewhere and knapping was especially focused on the renewal and manufacture of tools, which are particularly abundant at the site [39]. The retouched items display a great variety of types, including single side-scrapers, notches, denticulates and retouched flakes. The assemblage differs from other Levantine Mousterian sites in the high percentage (20%) of composite tools and very few points and naturally backed knives [39,47].

## Methodology

In this study we applied a comprehensive approach including technological and morphological study of archaeological material, systematic knapping experiments and microscopic use-wear analysis of the experimental and archaeological collections. Our experimental and use-wear study, besides having the goal of testing whether the marks on the bulb areas of the archaeological blanks are compatible with their use as retouchers, also aimed at the identification of different gestures that the knappers applied during retouching. Considering that most of the archaeological bulb retouchers are formal tools, such as Mousterian points and side-scrapers, use-wear analysis was also focused on detecting traces related to additional activities and reconstructing the functional history of the tools.

### Experiment

In the experiment conducted within this study, we focused exclusively on flint, because all the bulb retouchers from Nesher Ramla and Quneitra were made on this material. The experimental blanks were made of three different types of flint: high quality, fine-grained flint from Campanian Mishash Formation, the most common raw material found in Nesher Ramla assemblage, a fine-grained flint of Eocene age and a coarser flint type from Eocene age. The use of types of flint of different textures aimed at examining whether different flint qualities affected the pace at which the marks developed due to retouching.

**Fig 4:**
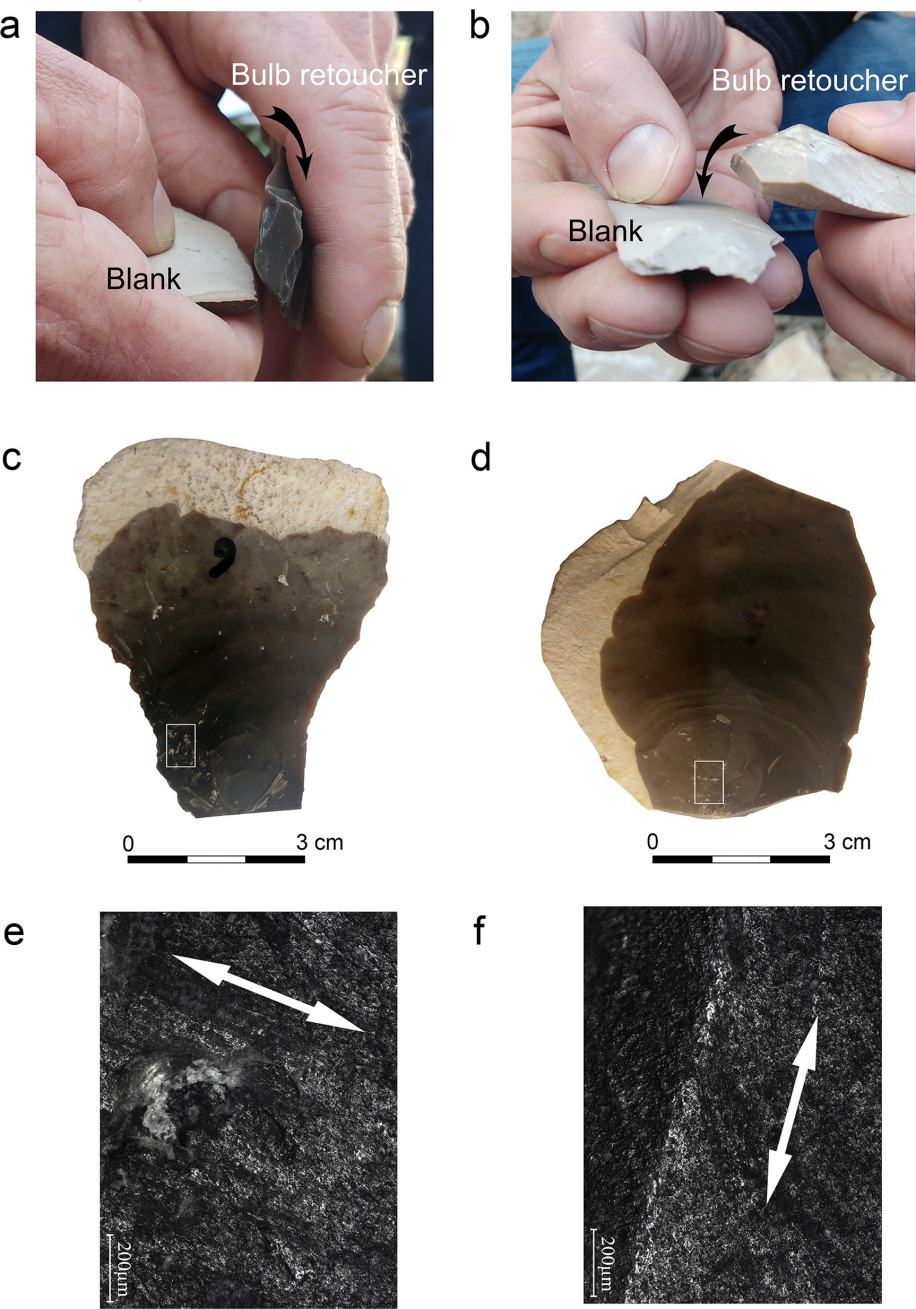
The retouching experiments and the resulting characteristic wear patterns. a: perpendicular retouching; b: parallel retouching; c: the retoucher used in the perpendicular mode showing traces on the left side of the bulb; d: the retoucher used in the parallel mode showing traces developed on top of the bulb; e: micrograph of striations and streaks of polish extending perpendicularly to the flaking axis of the blank (x100); f: striations extending parallel to the flaking axis of the blank (x100).

Six knappers with different skill levels took part in the experiment and were rated I—III (high ranking representing higher experience). Three knappers were regarded as being of level I, one as level II and one as level III. We deliberately choose knappers of different levels of expertise in order to establish if the formation of the retouching marks was affected by the knapper skill’s level. The knappers retouched several flint blanks using the bulb of percussion of flint flakes as retouchers. The latter were 26 flint flakes and blades of different weight and size. Two methods of retouching were employed during the experiment. In the first method (the ‘*perpendicular method’*), the retouchers (n = 9) were positioned with the bulb of percussion oriented perpendicular to the edge of the target blank (Fig 4A). In the second method (the ‘*parallel method’*), the knapper held the retouchers (n = 16) at the distal end, with the ventral face facing down and the bulb of percussion oriented parallel to the edge of the target blank (Fig 4B). The goal was to assess if different retouching motions would affect the location and type of impact marks. Each retoucher was used to manufacture one or two side-scrapers (S1 Fig). Our experiment focused only on the manufacture of side-scrapers, since the Nesher Ramla tool-kit is quite standardized and dominated by this type of tools. Side-scrapers are also among the most common tools found within Quneitra assemblage. In order to evaluate the possible extent of the bulb retouchers use-life, the number of retouching strikes was counted for each retouching session. Each retouched blank was measured and weighed before and after the retouching session.

### Archaeological material

Bulb retouchers were identified according to macroscopic features visible to the naked eye (i.e. pitting and crushing on the bulb of percussion area) during the sorting and techno-typological study of the lithic assemblage of Nesher Ramla and Quneitra. Although technically not bulb retouchers, four items with marks on the dorsal face (n = 4) and an item with marks on a fracture plane were also included in this study. It is of importance to note that, based on the experimental retouching, sessions of short duration did not leave any macroscopic trace on the bulbs’ surfaces. Consequently, it is possible that archaeological artefacts used only briefly as bulb retoucher might have been overlooked in the sorting procedure. This implies that the actual size of the bulb retouchers assemblages of the two sites is potentially larger than recognized by the naked eye. The active areas of bulb retouchers were described according to a specific attribute list especially developed for this study, including a characterization of both macro and microscopic features (S1 Text).

### Use-wear analysis

The current study integrates low power observations (using a stereomicroscope Nikon SMZ 745T at magnification of up to x50) to define traces on the macro-scale (such as edge removals, pitting and abrasions) and high power observations (using a metallurgical microscope Leica DM 1750M at magnification of x100-500) to define traces on the micro-scale (including polishes, streaks and striations). Traces were characterized based on attributes that define wears associated to use or prehension [49,50], while the functional reconstruction was based on the comparison between traces observed on the archaeological and experimental items. The experimental program focused on replicating the marks observed on the archaeological bulb retouchers, while for the identification of other kinds of use-wears we used the experimental tools of the reference collection at the Use-Wear Analysis Laboratory at the Zinman Institute of Archaeology, University of Haifa. This collection comprises tools that were used in various activities and bearing diagnostic traces, including those produced by the contact with various materials such as bone, flesh and wood, and those produced through hafting and prehension.

## Results

### Experiment results

In our experiment we observed that macroscopic marks caused by retouching usually begin to appear in low numbers after at least 100 or even 200 strikes, which were enough to produce a side-scraper with a continuous scalar retouch of ca 50 mm of length. We noticed that, in most cases, short retouching sessions (50–100 strikes) did not result in any macroscopic marks. In our experiment the degree of expertise of the knapper didn’t affect the way marks were produced, as both experienced and unexperienced knappers produced similar set of marks, at similar pace. Knappers of all levels of expertise mostly created only sparse marks even after 200–300 strikes, and in few cases, did not produce any mark visible to the naked eye even after more than 150 strikes. We found however a correlation between the texture of the flint and the pace of development of the marks: the pits developed quicker on the fine grained, brittle flint of the Mishash Formation and on the fine-grained Eocene flint (on average 130 strikes) than on the coarse-texture Eocene flint (on average around 240 strikes). In our experiment similar impact marks were produced on both types.

Using bulb retouchers we were able to produce side-scrapers characterized by a regular, invasive retouch, similar to that of Nesher Ramla side-scrapers. As our experiment goal was to produce one or two side-scrapers, our retouching sessions stopped between 200–350 strikes maximum. Although at this stage we obtained few marks similar to the archaeological ones, we never replicate the deep crushing and superimposed traces identified on some of the archaeological specimens which appear to have been intensely utilized.

### Macro- and micro-wear analyses of experimental bulb retouchers

**Fig 5:**
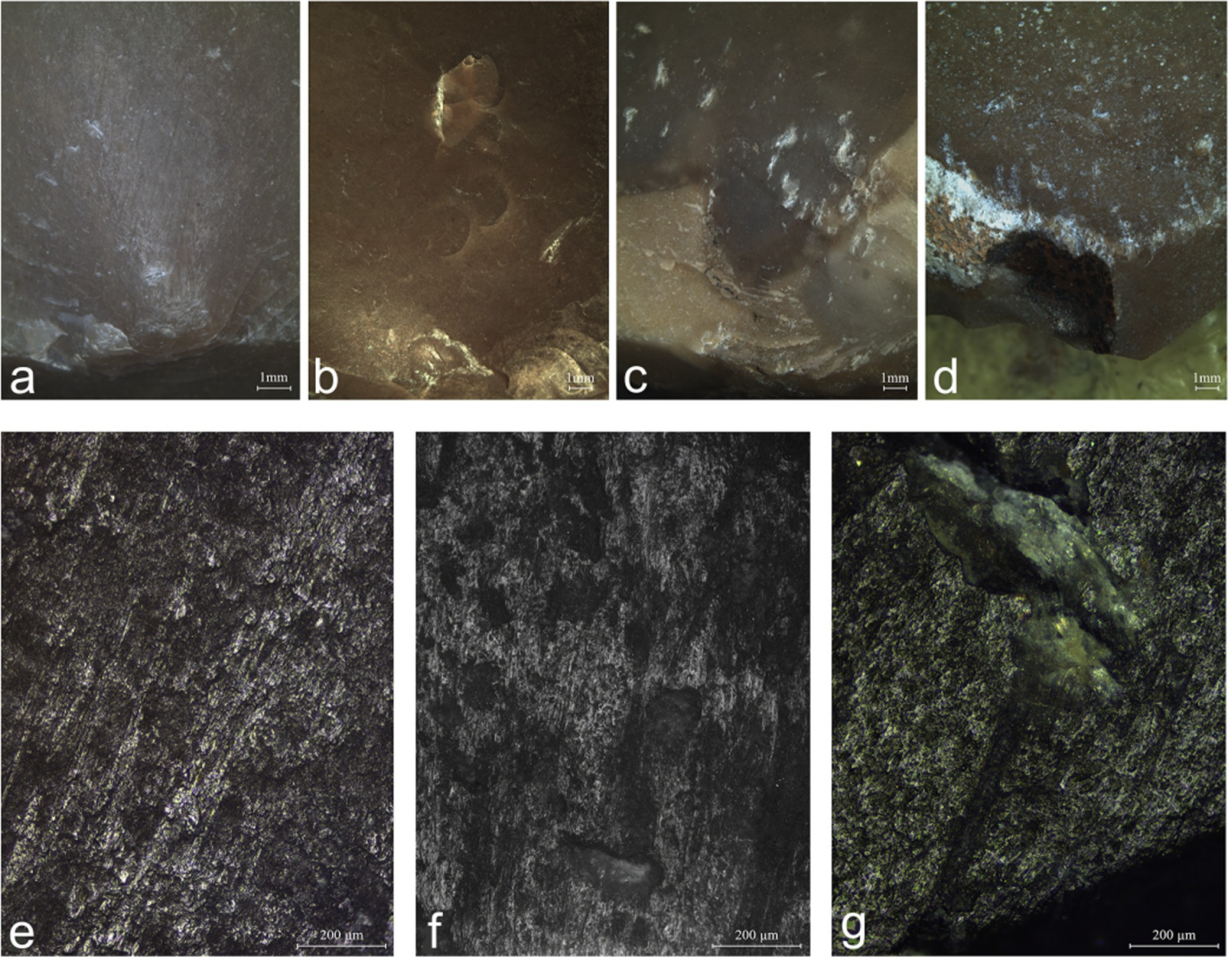
Experimental use-wear. Macrographs (a-d) and micrographs (e-g) of wear patterns observed on the experimental tools. a: parallel retouching after 436 blows showing elongated pits associated with streaks of polish visible to the naked eye (x10); b: parallel retouching after 110 blows showing incipient cones (x6.7); c: perpendicular retouching after 150 blows showing the characteristic elongated pits with similar orientation (x6.7); d: abrasion of the edge of the blank associated with pitting (x20); e: parallel retouching striations extending in two main directions indicating slight change in the orientation of the retoucher (x100); f: parallel retouching dull, rough and striated polish indicating the direction of the motion of the retoucher parallel to the flaking axis of the retouched blank (x100); g: perpendicular retouching rough polish associated with an elongated pit and a wide groove (x100).

The use-wear analysis of 26 experimental bulb retouchers highlighted several types of macroscopic and microscopic traces produced by the retouching (Fig 5). The main type of marks identified on the bulbar surface of the experimental retouchers is the pitting, defined as the fracturing of the flint surface at the point of impact, usually visible to the naked eye. Polish and linear features are associated with the pitting, sometimes visible under low magnification (ca. x20) but better discernible under high magnification (x100-200). An additional type of wear is the abrasion, developed on the edges of the retoucher.

**Fig 6:**
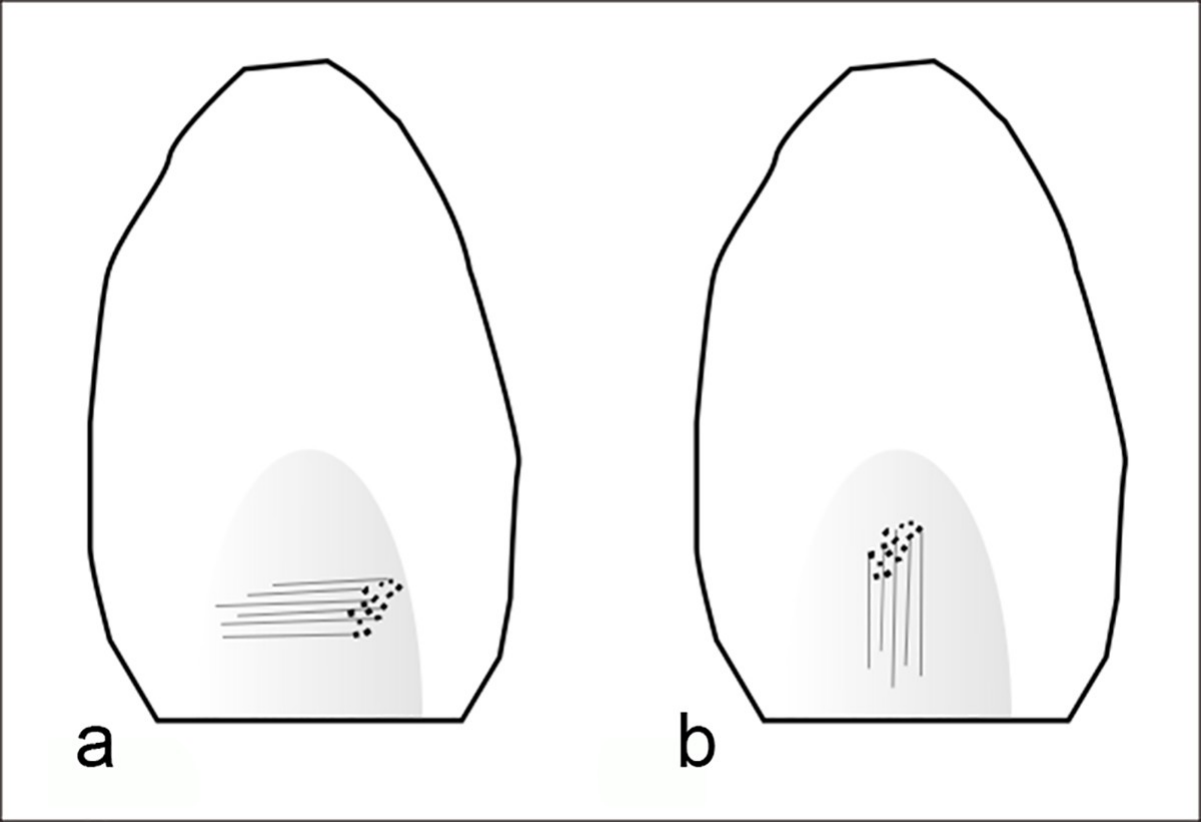
Schematic representation of the location and orientation of wears originating from different retouching modes. a) perpendicular retouching mode b) parallel retouching mode.

**Pitting** is the most diagnostic wear produced during the experiments. The pits are usually elongated with a clear long axis (n = 20; Fig 5C). In some cases, the pits were also amorphous (n = 10), varying in size, depth and quantity. In few occasions, the fracturing of the bulb resulted in isolated incipient cones (n = 7; Fig 5B). The number of pits developed on the surface is proportional to the intensity of use. When pits did not develop (n = 2), the use as retoucher was identified through other characteristic marks, including abraded edges at the margins of the retoucher (Fig 5D) or polish and linear features (Fig 5E–5G). The experiment showed that the location of the pits reflects the mode of retouching (Table 1 and Fig 6). In the parallel retouching mode, pits appear on top of the bulb or in its distal part (Table 1), while in the perpendicular mode pits appear mostly on the bulb sides (Table 1).

**Table 1:** Location and orientation of wears originating from different retouching modes on the experimental retouchers.

		Parallel mode		Perpendicular mode	
		n	%	n	%
**Location of pits**	On the bulb or on the distal part of the bulb	9	56	2	22
** **	On the bulb slopes	5	31	7	78
** **	No pits	2	13	-	-
	Total	16	100	9	100
**Location of linear features**	On the bulb	14	88	4	44
** **	On the slopes of the bulb	-	-	4	44
** **	No streaks	2	13	1	11
	Total	16	100	9	100
**Orientation of linear features in relation to the flaking axis of the tool**	Perpendicular	2	13	7	78
** **	Parallel	10	63	-	-
** **	Perpendicular and parallel	1	6	-	-
	Oblique	1	6	-	-
	Sparse	-	-	1	11
	No streaks	2	13	1	11
	Total	16	100	9	100

**Linear features** are of three types: the first and the most common type is a long, shallow and narrow striation (Fig 5E), appearing on all experimental tools, except for one (n = 25). The second type is a long, deep and wide groove, which was detected occasionally (n = 4), in combination with the former. Linear streaks of polish also appear occasionally (n = 8), associated with the striations and grooves. These features are the result of the contact between the retoucher and loose flint particles that are released during the impact and dragged along the retoucher surface. Linear features can thus show the direction of movement of the retoucher against the blank, therefore indicating the retouching mode (parallel or perpendicular). In the parallel retouching mode, the linear features appear on the bulb, extending parallel to the flaking axis of the retoucher, while, in the perpendicular mode, they appear on the bulb and its sides, extending perpendicular to the flaking axis of the retoucher (Table 1 and Fig 6).

**Polish** on the bulb was occasionally observed (n = 10) on retouchers that were used for the higher number of strikes (147–336 strikes). Under high magnification the polish appears rough in texture, bright and pitted (Fig 5F). Since the polish is spread and shows no directionality, it can easily be mistaken for post-deposition surface modifications (PDSM) on archaeological pieces, especially because the protruding surface of the artefacts bulb is the most vulnerable to post-depositional processes. However, when the polish appears in combination with the pitting and striations, it is possible to differentiate it from PDSM.

**Abrasion** is a worn crushed edge located on the proximal edge of the butt in the parallel mode of retouching, and on the side edge of the retoucher in the perpendicular mode (Fig 5D). A large part of the experimental retouchers showed signs of abrasion (44%; n = 12).

### The bulb retouchers of Nesher Ramla

**Fig 7:**
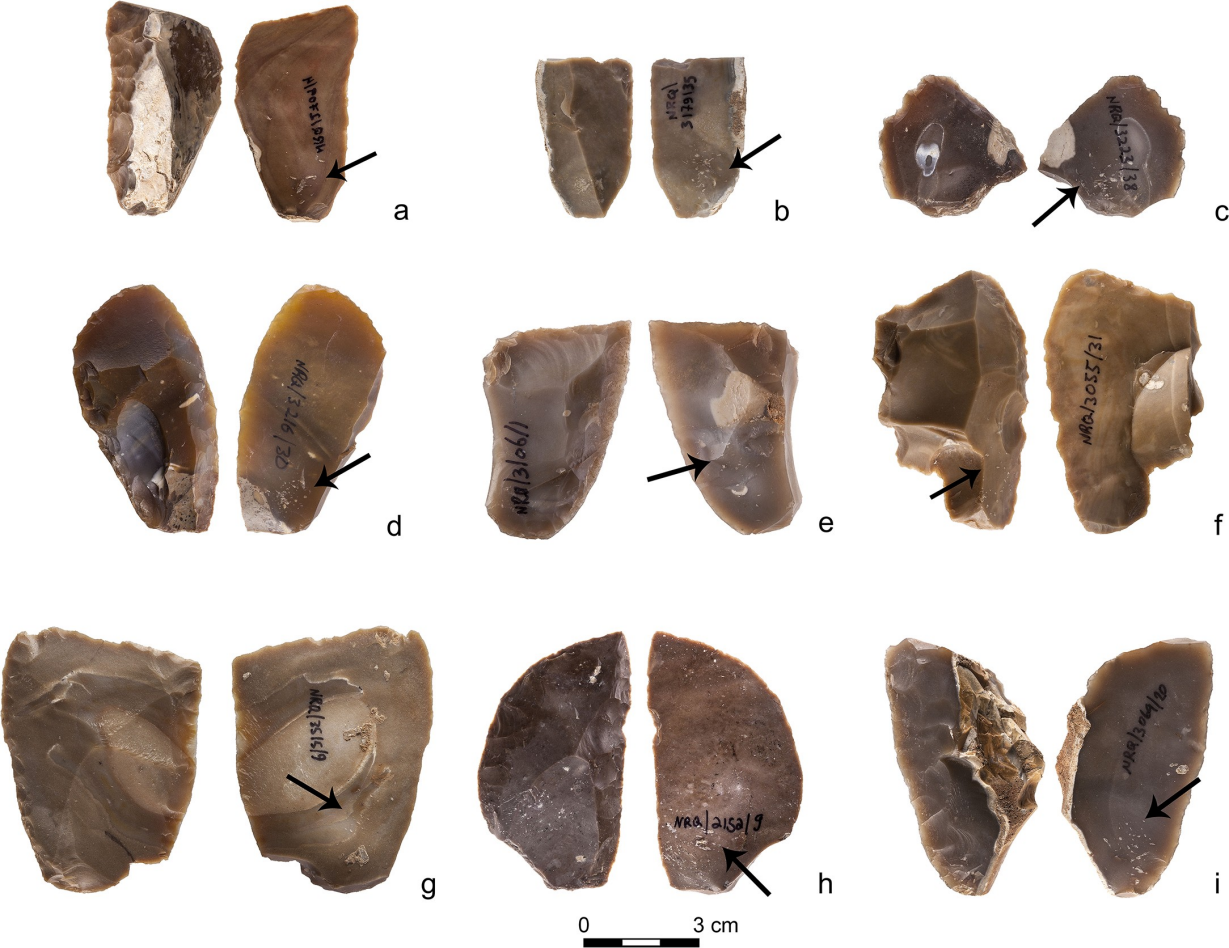
Some examples of bulb retouchers from Nesher Ramla. Arrows point to the retouching marks (Photos by Tal Rogovski).

The bulb retouchers assemblage from Nesher Ramla is the largest documented so far. It comprises 159 items retrieved from four stratigraphic units (I, IIA, IIB and III; Fig 7). Most of the bulb retouchers originate from unit IIB (n = 120), while unit IIA and III yielded 14 and 17 bulb retouchers respectively, and only 8 were identified in unit I (Table 2). Marks appear on the central part of the bulb (n = 94), its distal part (n = 31) or on its slopes (n = 25), sometimes in the middle of the ventral face (n = 7).

**Table 2:** Frequencies of bulb retouchers in the total lithic assemblage of each archaeological unit.

Unit	Bulb retouchers		Retouched pieces		Intensely retouched convergent tools	
	n	%	n	%	n	%
**I**	8	0.2	315	7.7	18	5.7
**IIA**	14	0.3	276	6.8	21	7.6
**IIB**	120	0.6	2,485	12.1	253	10.2
**III**	17	0.2	1,226	11.0	43	3.5

Bulb retouchers frequencies are compared to frequencies of retouched pieces and of intensely retouched convergent tools (convergent and *déjeté* side-scrapers, Mousterian points). Frequencies of retouched pieces are calculated on the total lithic assemblage (> 2 cm); frequencies of intensely retouched convergent tools are calculated on the total assemblage of retouched pieces. A positive correlation is highlighted between frequencies of bulb retouchers and intensely retouched convergent tools (r = 0.90; p = 0.09).

#### Blank selection

The most striking feature of the bulb retouchers assemblage from Nesher Ramla is the almost exclusive selection of retouched items to be used in retouching activities (Table 3). The few unretouched blanks are naturally backed knives (n = 6), cortical elements (n = 3), core trimming elements (CTEs; n = 1), Nahr Ibrahim pieces (n = 2), core on flakes (n = 1) and flakes (n = 3).

**Table 3:** Typological breakdown of bulb retouchers and of lithic artefacts in Nesher Ramla and Quneitra.

Typology	Nesher Ramla				Quneitra			
	Bulb retouchers		Total assemblage		Bulb retouchers		Total assemblage	
	n	%	n	%	n	%	n	%
Simple side-scrapers	63	44	1,686	39	2	18	445	14
Double side-scrapers	8	6	202	5	1	9	145	5
Convergent side-scrapers	14	10	135	3	2	18	106	3
*Déjeté* side-scrapers	6	4	93	2	-	-	54	2
Transversal side-scrapers	1	1	89	2	1	9	79	2
Other side-scrapers	4	3	29	1	-	-	126	4
Mousterian points	29	20	156	4	-	-	33	1
Retouched Levallos points	3	2	69	2	-	-	11	-
Retouched flakes and blades	5	3	659	15	1	9	228	7
Notches and denticulates	2	1	164	4	4	36	933	29
Other tools	2	1	489	11	-	-	1,051	33
Broken tools	6	4	516	12	-	-	ind	ind
**Total retouched pieces**	**143**	**90**	**4,302**	**11**	**11**	**85**	**3,211**	**38**
Non-retouched NBKs	6	4	2,909	7	-	-	26	-
Other non-retouched blanks	10	6	32,739	82	2	15	5,167	61
**TOTALS**	159	100	39,950	100	13	100	8,404	100

For typological breakdown in each archaeological unit of Nesher Ramla see S3 Table.

**Fig 8:**
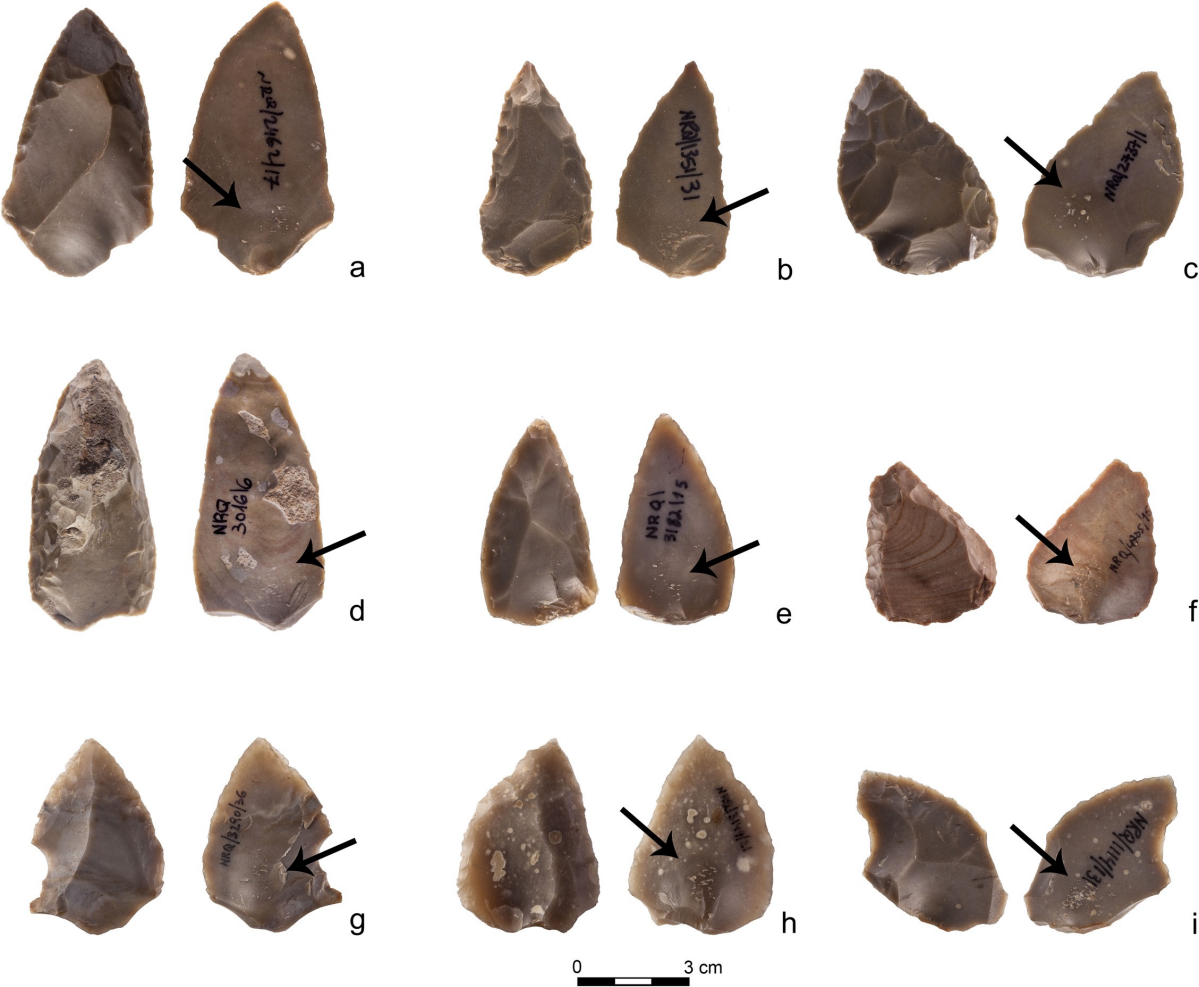
Convergent shaped bulb retouchers from Nesher Ramla. a-b-d-e-f- Mousterian points; c- intensely used bulb retoucher on a *déjeté* side-scraper (Photos by Tal Rogovski).

Simple side-scrapers, the most common tool group found within the complete tool assemblage, are also best represented among the bulb retouchers (Table 3). Mousterian points are the second largest blank group (20%). The latter sharply contrasts with the overall assemblages of retouched pieces at the site, where Mousterian points range between 0% (Unit III) and 5% (Unit IIB and IIA; Table 3 and S1 Table). The bulb retouchers assemblage is characterized by high frequencies of intensely retouched artefacts with a sub-triangular, convergent shape—i.e. Mousterian points, convergent and *déjeté* side-scrapers (Table 3 and Fig 8). Tools with a convergent shape are, on the other hand, much less common among the retouched pieces. According to some scholars [51,52], these tool forms are the most extensively re-sharpened through retouch and with the longest use-lives among Middle Palaeolithic types. There is a positive correlation between the occurrence of bulb retouchers along the sequence and the presence of intensely retouched convergent tools (r = 0.90; p = 0.09; Table 2). In contrast, tools with light, non-invasive retouch, such as retouched flakes and blades, are underrepresented in the bulb retouchers assemblage (Table 3 and S1 Table).

#### Bulb retouchers raw material composition

Most lithic artefacts at Nesher Ramla were knapped from nodules and pebbles of high quality flint from the Mishash Formation, the nearest outcrop of which is located only few hundred meters away from the site. Sources for other types of flint are located at distances greater than 15 km from the site (i.e., non-local sources), except for a small outcrop of Eocene flint located some 5 km south to the site. Almost all lithic artefacts that are made on non-local flint are formal tools, most of them intensely retouched (S2 Table). The bulb retouchers assemblage is composed mainly of artefacts made on non-local raw materials (i.e. non-Mishash flint). The differences in raw material composition between the bulb retouchers and the total tool assemblage are significant. While 37% of the formal tools are made on non-local raw material, more than 50% of the bulb retouchers assemblage are made on such raw materials (Table 4). These data suggest that blanks used as bulb retouchers were manufactured in other locations and transported to the site, where they were abandoned.

**Table 4:** Raw material partition (Mishash-local; non-Mishash-unlocal) within the different archaeological units of Nesher Ramla.

Unit	Raw material bulb retouchers						Raw material retouched pieces						Raw material total assemblage					
	Mishahs		non-Mishash		Total		Mishash		non-Mishash		Total		Mishash		non-Mishash		Total	
	n	%	n	%	n	%	n	%	n	%	n	%	n	%	n	%	n	%
**I**	4	50	4	50	8	100	108	71	43	29	151	100	1,234	88	176	12	1,410	100
**IIA**	4	29	10	71	14	100	60	63	35	37	95	100	836	85	145	15	981	100
**IIB**	60	50	60	50	120	100	818	62	496	38	1,314	100	6,476	81	1,485	19	7,961	100
**III**	7	41	10	59	17	100	782	64	444	36	1,226	100	7,922	71	3,179	29	11,101	100
**TOT**	75	47	84	53	159	100	1,768	63	1,018	37	2,786	100	16,468	77	4,985	23	21,453	100

* Raw material identification was carried on only on complete lithic artefacts.

#### Use wear study of Nesher Ramla archaeological material

**Fig 9:**
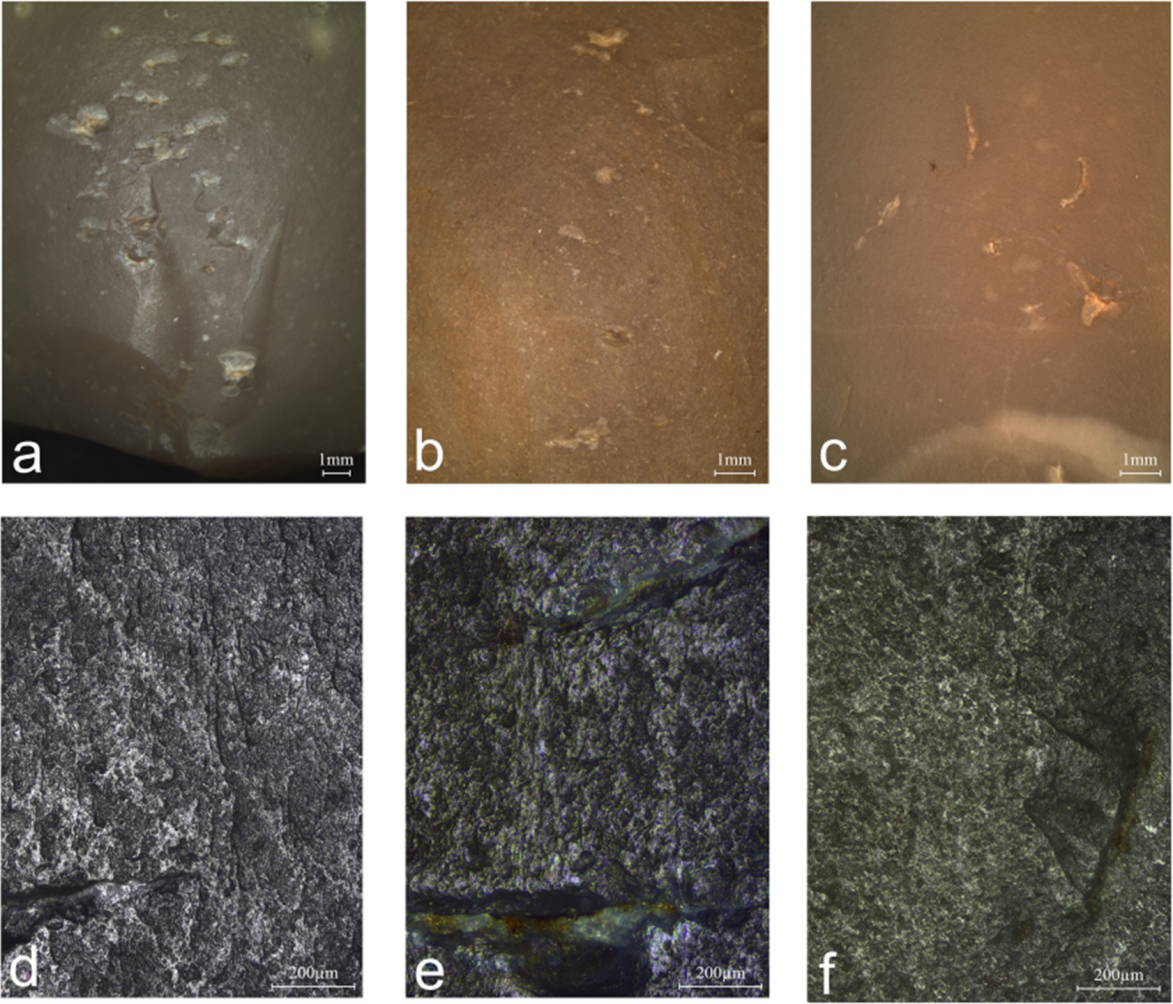
Retouchers use-wear patterns showing the retouching mode and the life history of the tools. a: Mousterian point (3149–21) with massive pitting on the bulb indicating a parallel retouching mode (x6.7); b: linear distribution of pits (x10); c: sparse distribution of pits (x10); d: streaks of polish oblique to the long axis of a pit, possibly indicating a perpendicular retouching mode (x100); e: polish and striations parallel to the long axis of the pit indicating a parallel retouching mode (x100); f: striations associated with a pit on a surface with no PDSM (x100).

Use-wear analysis on a sample of 142 archaeological items revealed marks comparable to the ones obtained through the experimentation (Fig 9). Pitting, detected on all the tools, is usually of the typical elongated type, occasionally combined with the amorphous type or with incipient cones, except for two cases where the marks were represented only by incipient cones. Pitting usually appears in dense clusters (n = 74; Fig 9A), in a linear spread (n = 42; Fig 9B), or sparsely in low numbers (n = 43; Fig 9C). The pitting marks on the archaeological items were usually combined with linear features (Fig 9E and 9F), streaks of polish or polish, observed under high magnification (x100-200), with the exception of some artefacts (n = 34) that bear only pitting. Furthermore, abrasion at the edge of the retoucher was far less common in Nesher Ramla material (7%; n = 11) than in the experimental one (44%; n = 12). These traces usually appear as a combination (e.g. pits with linear features and polish), however linear features are the most dominant elements appearing in relation to the pits.

Based on the experimental results, the distribution of the traces was used to infer the retouching mode (Table 1, Fig 6). When the orientation of the linear features (the most diagnostic wears related to the mode of use) and at least one of the other traits (location of pits and linear features) pointed to a specific retouching mode, artefacts were categorized as being used parallelly or perpendicularly (Table 5). The other bulb retouchers were classified as not diagnostic.

**Table 5:** Representation of the two different retouching modes within Nesher Ramla archaeological sample.

Mode of retouching	I		IIA		IIB		III		Total	
	n	%	N	%	n	%	n	%	n	%
**Parallel mode**	1	13	2	14	21	18	4	24	28	18
**Perpendicular mode**	1	13	1	7	11	9	3	18	16	10
**No use-wear study**	-	-	-	-	17	14	-	-	17	11
**Not diagnostic**	6	75	11	79	71	59	10	59	98	62
**TOTAL**	8	100	14	100	120	100	17	100	159	100

In some instances, linear features were oriented oblique to the tool axis (n = 27), a case that we encounter only once in the experimental material: these retouchers were classified as belonging to the perpendicular or parallel method exclusively on the basis of the location of the pits and/or linear features (note their orientation). Therefore, the classification of the mode of retouching of these artefacts was considered as “low certainty”, because it is possible that oblique linear features may be the result of both retouching methods. In addition, abrasion, a common feature in the experimental material, rarely appeared in the archaeological sample (n = 11): the small number of artefacts bearing abrasion prevented a thorough correlation between abrasion position and mode of retouching.

These differences between the archaeological and experimental samples may be the result of the more controlled environment of the experimental program, where knappers focused on maintaining the same orientation of the retouchers along all the retouching performance. Nesher Ramla inhabitants, on the other hand, might have changed the angle of the retoucher within the same retouching session or could have used the same retoucher in different retouching episodes (with slightly different gestures each times). It is also possible that different knappers could have used the same retoucher through time.

**Fig 10:**
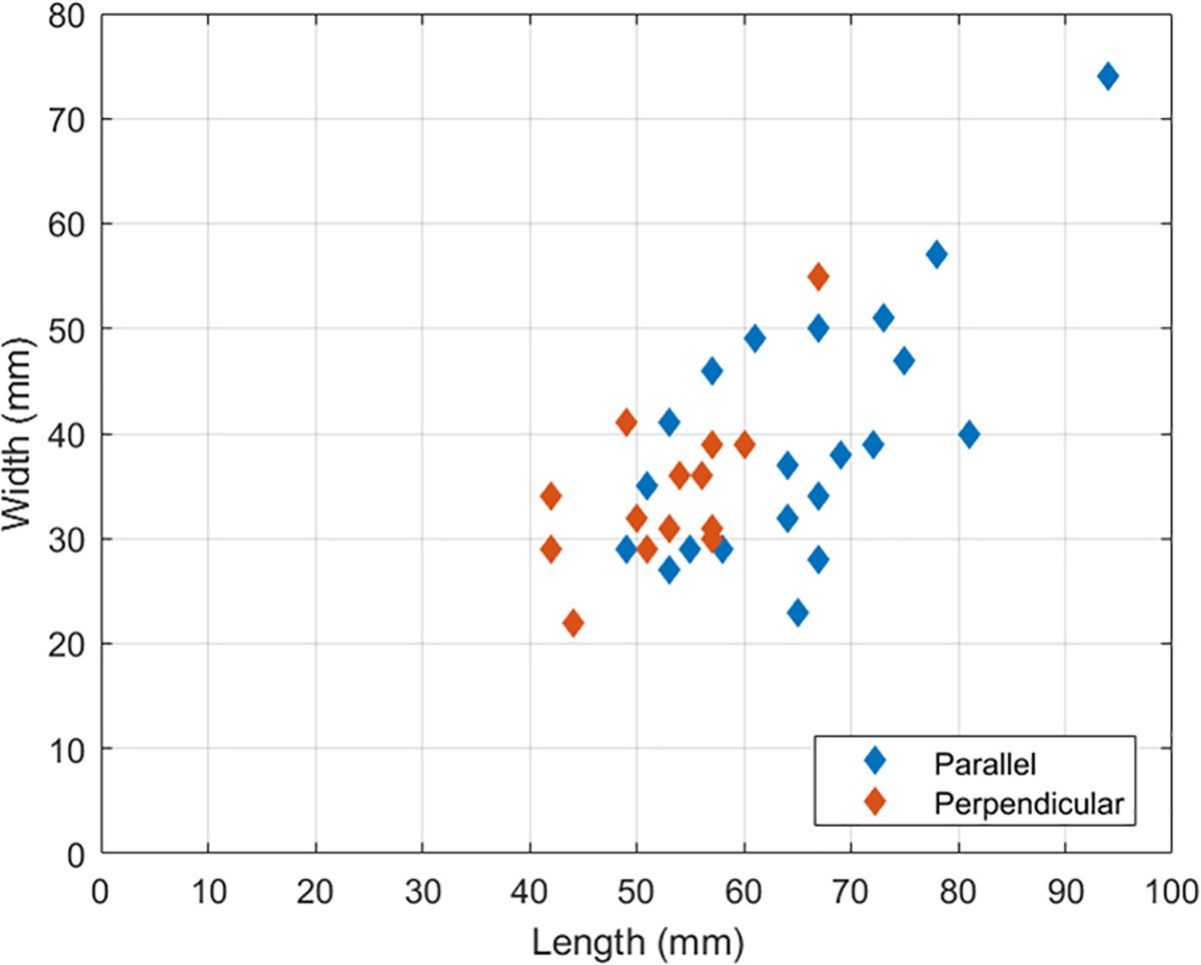
Length and width of retouchers used in the parallel and perpendicular modes at Nesher Ramla. Bulb retouchers used in the perpendicular mode (in red) appear more homogeneous in size and shorter compared to the parallel ones (in blue). Note that the width is similar for both bulb retouchers categories.

Forty-four bulb retouchers were classified as diagnostic in terms of retouching mode. The results show that the parallel mode is more common than the perpendicular one within all the studied assemblages (64% and 36% of the diagnostic bulb retouchers respectively). Retouchers used in the parallel mode are longer and heavier than those used in the perpendicular mode (Table 6, Fig 10 and S1 Data). The latter also show less size variability within the assemblage (Table 6 and Fig 10). The Wilcoxon rank sum test (U test) shows that the differences in length and weight are statistically significant (p = 0.002 and p = 0.024 respectively). However, the two retoucher categories do not show significant variation in thickness and width (p = 0.144 and p = 0.860 respectively).

**Table 6:** Metric attributes of the bulb retouchers from Nesher Ramla and Quneitra.

	Nesher Ramla perpendicular-mode bulb retouchers		Nesher Ramla parallel-mode bulb retouchers		Nesher Ramla total bulb retouchers assemblage		Quneitra total bulb retouchers assemblage	
	average	SD	average	SD	average	SD	average	SD
**Maximum length (mm)**	52.8	7.1	64.9	11.1	59.6	11.7	49.4	12.8
**Axis length (mm)**	50.0	9.7	63.8	11.3	57.2	12.6	45.8	15.1
**Axis width (mm)**	36.6	8.1	38.8	12.4	38.9	9.1	38.1	7.6
**Thickness (mm)**	9.4	2.7	11.4	4.1	10.3	3.6	10.5	3.1
**Weight (mm)**	16.5	4.1	35.9	26.9	27.2	17.7	-	-

Parallel and perpendicular bulb retouchers, not including items classified as “low-certainty”.

Bulb retouchers used in the parallel mode appear longer also when artefacts classified as “low certainty” are included in the sample (p = 0.023). This might suggest that the classification based only on the location of pits, polishes and linear features (but not their orientation) is effective in distinguishing between the two retouching modalities.

**Fig 11:**
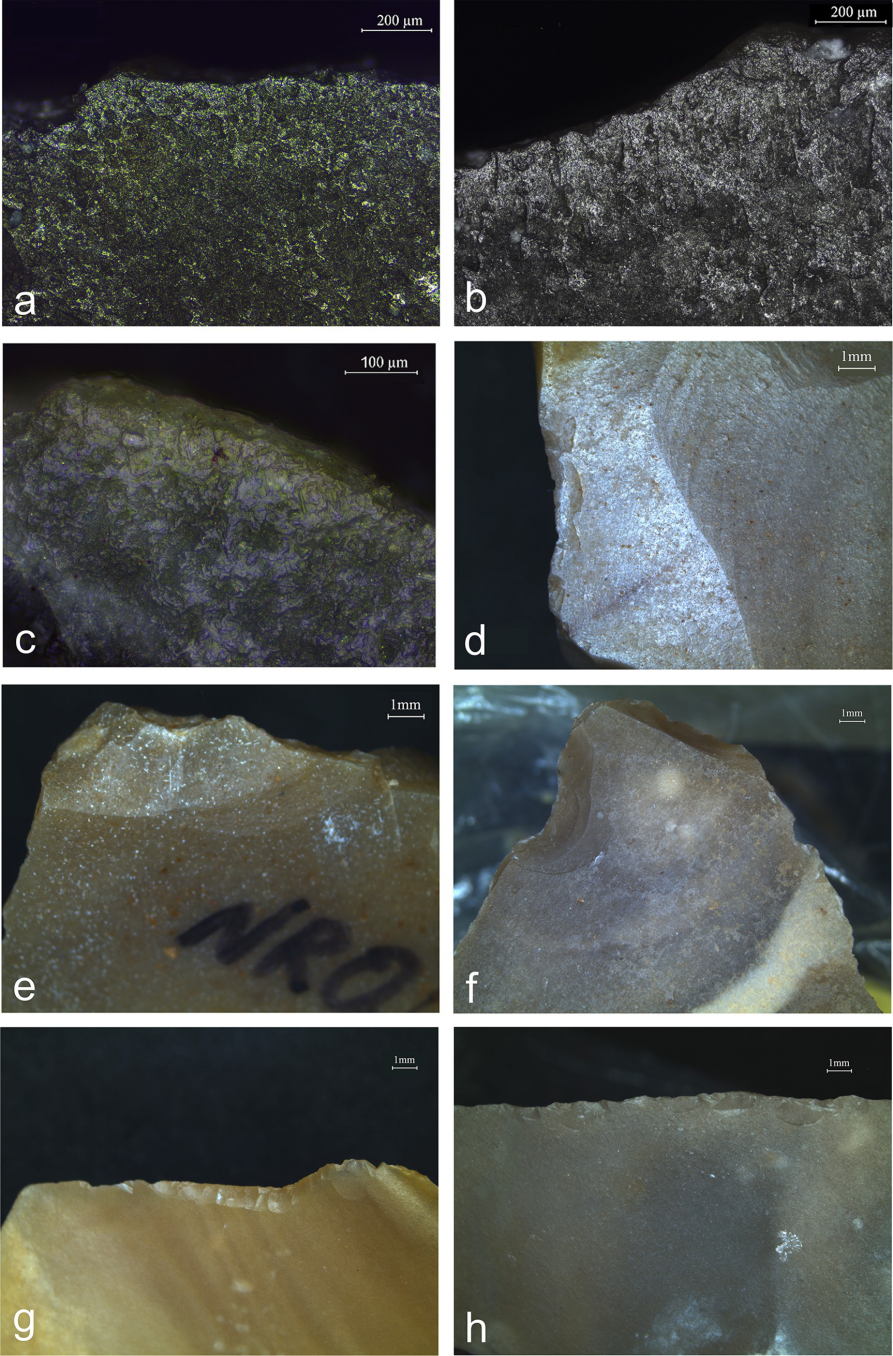
Use-wear traces identified on Nesher Ramla retouchers edges. a: butchering traces including meat cutting and occasional contact with bone on the edge of a simple side-scraper (x100); b: meat cutting polish associated with butchering activity identified on a denticulate edge (x100); c: edge rounding, occasional short deep striations and faint polish associated with meat scraping of a bone on the edge of a side-scraper (x200); d: fracture associated with hafting on the right lower side of a *déjeté* side-scraper (x10); e: impact fracture on the tip of a *déjeté* side-scraper (x10); f: impact fracture on the tip of a Mousterian point (x6.7); g: edge removals indicating scraping of a hard material on the edge of a simple side-scraper (x10); h: edge removals produced by scraping of hard material on the edge of a simple side-scraper (x10).

Considering that most of the retouchers are formal tools, one of the goals of use-wear analysis was to verify if these blanks were used in other tasks besides the retouching. Use-wear analysis revealed wear traces on 21% (n = 30) of the analysed bulb retouchers (Table 7), while the rest of the assemblage show high post-deposition surface modifications that prevented identification of other use-wear traces. The identified traces are related to different type of activities, such as plant and wood-working, hunting and carcasses processing (Fig 11). The various activities appear equally represented, but the small sample size prevented the recognition of specific patterns. In all cases, the wear traces appear on the tool edges, separated from the retouching marks, which appear on the bulb area of the blanks. It was therefore impossible to determine the sequence of the different actions.

**Table 7:** Use-wear traces observed on Nesher Ramla bulb retouchers assemblage.

Activities	n
Bone and flesh scraping	1
Bone scraping	1
Cutting flesh with contact with bone	1
Flesh cutting	1
Scraping flesh off bone	1
Butchering	1
**Total butchering related activities**	**6**
Plant scraping	3
Plant working	3
Wood scraping	1
Tuber extraction	1
**Total plant working tools**	**8**
Cutting hard material	1
Digging tool	1
Scraping soft material	4
Scraping medium hardness material (plant?)	1
Scraping hard material	1
Scraping material not diagnostic	2
Hafting traces	2
Hafting traces associated to impact fracture	2
Impact fractures	2
Not diagnostic	3
No traces	24
PDSM	85
No use-wear study	17
**Total**	**159**

#### Nesher Ramla bulb retouchers use-lives

**Fig 12:**
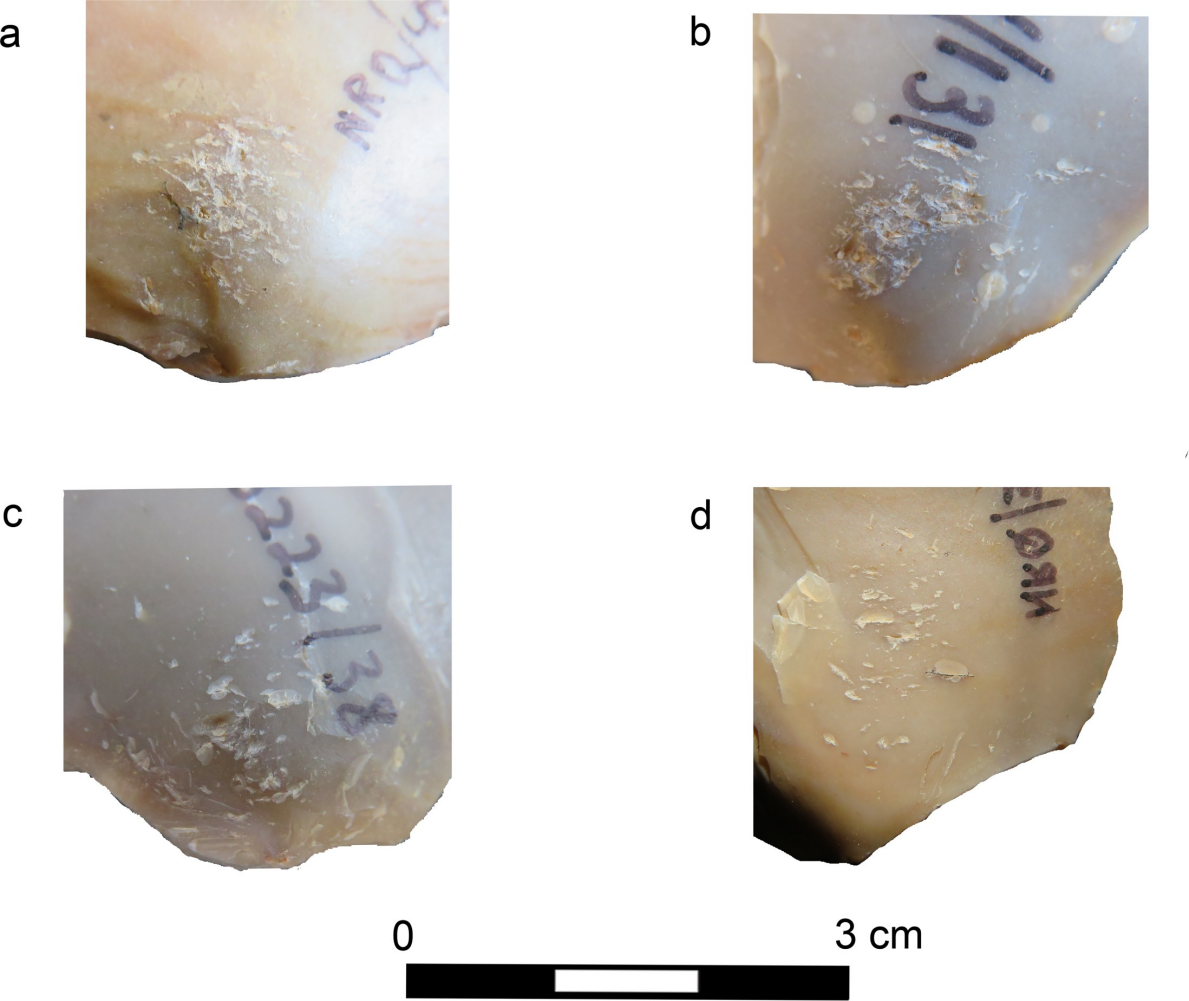
Examples of bulb retouchers from Nesher Ramla exhibiting intense use. a- high density of pits on the bulb of a Mousterian point; b-superimposing pits and deep crushing on the bulb of a *déjeté* side-scraper (see Fig 7C); c- pits and crushing on the bulb of a simple convex side-scraper; d- high density of pits on the bulb of a Mousterian point.

As inferred from the experimentation, marks start to appear in low density after at least 100–200 strikes, enough to create a continuous scalar-retouched edge ca 50 mm long. In our experiment we never obtained highly dense marks, with deep crushing and clusters of superimposed pits (> 30) similar to the ones visible on some of the archaeological specimens (17% of the bulb retouchers; n = 27; Fig 12). This might suggest that some archaeological bulb retouchers were used more intensely than the experimental ones before being discarded. The other bulb retouchers of Nesher Ramla exhibit medium intensity of marks (32%; n = 51), or only sparse marks (49%; n = 78), more similar to the one obtained during our experiment. Few other artefacts (n = 5) exhibit two separate clusters of marks on the ventral surface, ventral and dorsal surfaces, ventral surface and striking platform. We suggest that these retouchers were used during at least two separate retouching sessions. In few cases, it was clear that the use as retoucher was followed by re-sharpening or retouching, before discard. These specimens (n = 9) are considerably smaller than the other bulb retouchers: their sizes would have prevented their use as retouchers since it would have been impossible for the knapper to hold them without covering the bulb of percussion area with his/her hand (Fig 7C).

**Fig 13:**
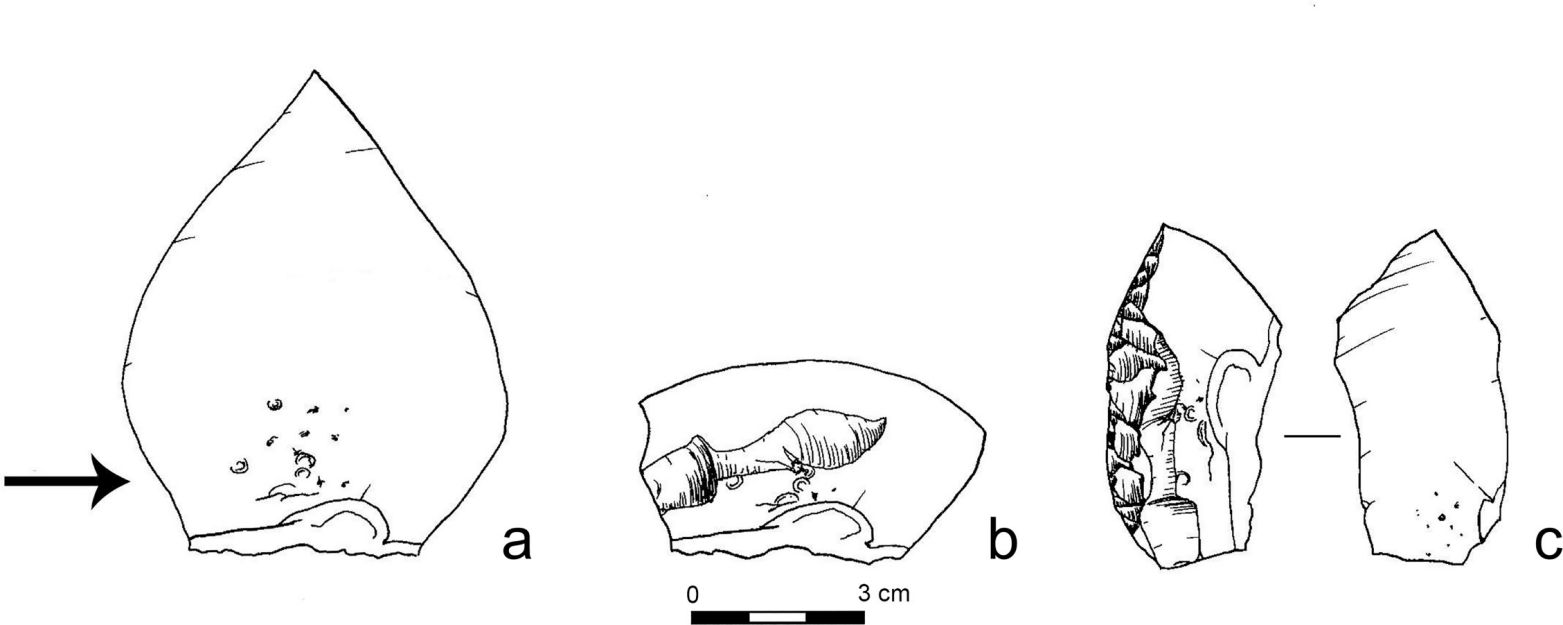
Bulb retoucher on a Kombewa flake with a long use-life. a: a big flake is used as bulb-retoucher; b: from the ventral face of the flake are extracted two consecutive flakes, the first one removes the area with the pitting marks; c: the second flake extracted is retouched into a side-scraper and used as bulb retoucher.

Two bulb retouchers are of particular interest: one is a Kombewa flake that exhibits at least two separate episodes of use as a retoucher on both surfaces. The blank was struck from a big flake, which was, in a previous stage, used as retoucher (Fig 13). The percussion marks on the bulb of the parent flake are still visible on the dorsal face of the Kombewa flake. Subsequently, the Kombewa flake was retouched into a simple side-scraper and used as retoucher, as attested by the pitting marks on the bulb (Fig 13). This implement attests for a complex use-life, where a blank was used as a retoucher (Fig 13A), later as a core (Fig 13A and 13B), a tool (Fig 13C) and again as a retoucher (Fig 13C) in a long sequence of actions.

**Fig 14:**
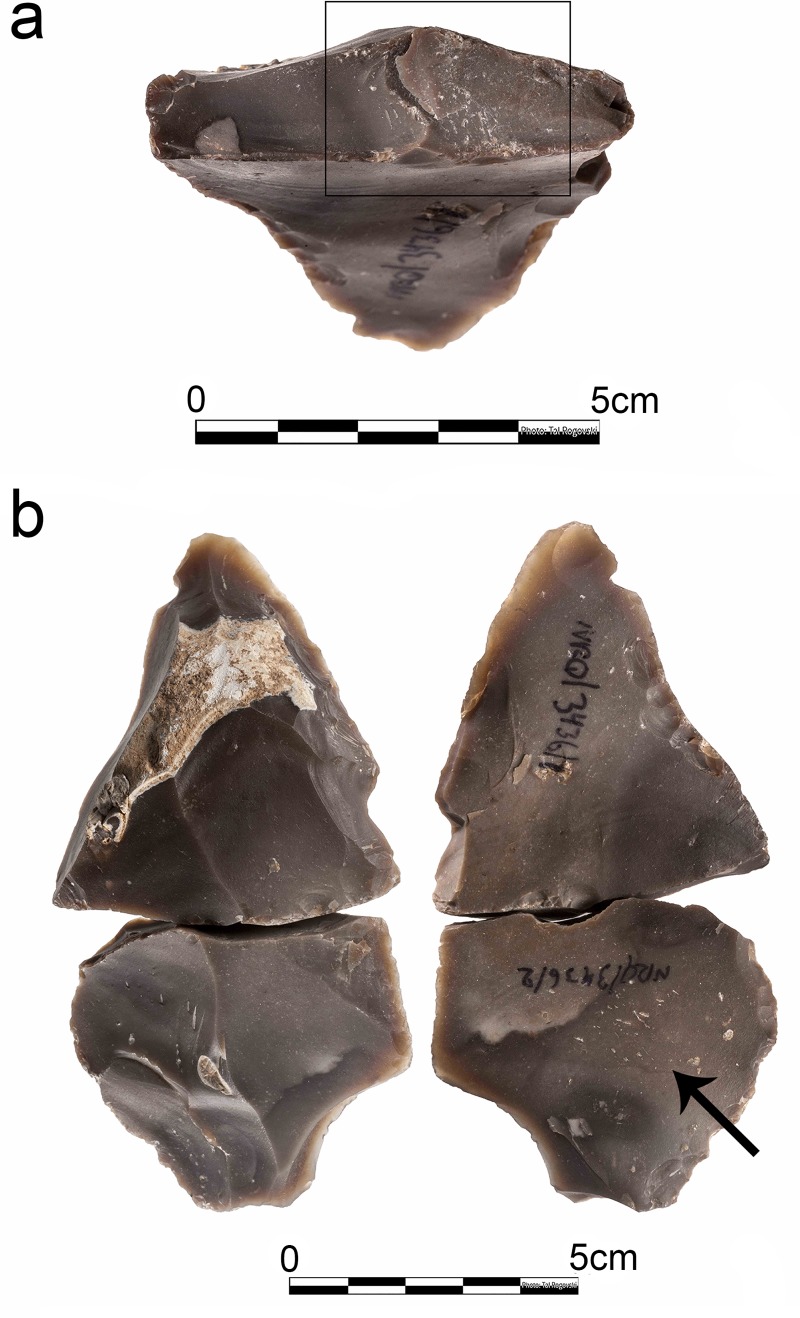
Blade used as retoucher (Unit IIB). The bulbar area was used as retoucher, subsequently the blade broke in two. The proximal fragment of the blade was further modified into a side-scraper (b). The distal fragment of the blade was used as retoucher too, the marks appear on the fracture surface (a). (Photos by Tal Rogovski).

Another interesting example is a side-scraper on a thick blade, that was used as a retoucher and later broke in two conjoinable parts. The proximal part, bearing the retouching marks, could not have been used as retoucher after the breakage because of its reduced dimensions. This proximal segment was subsequently retouched again: the retouch partially covers the fracture. The distal fragment of the blade was used as a retoucher as well, exhibiting marks on the surface of fracture. It is the only blank that exhibits retouching marks on a break (Fig 14). The peculiar position of the marks attests that the item was used as a retoucher after the original blank was broken.

In three other cases, bulb retouchers obtained on previously retouched or unretouched blanks were turned into core-on-flakes, where the removed blank negatives cut into the retouching marks, attesting to a final *débitage* stage for these implements. In these cases, the function of the blank changed irreversibly, as the reduced dimensions of the blanks prevented their further use as retouchers, or because the convexity offered by the bulb of percussion was exploited for blank production. Thus, bulb retouchers appear to have been used as multi-purpose, versatile implements, that changed and reprised their role several times before being abandoned.

### Quneitra material

The bulb retouchers discovered at Quneitra (n = 13) originated from Area B of the excavation and are all made on non-local flint, attesting to long-distance transportation [48]. The macroscopic marks observed on the Quneitra material, mainly elongated pits associated with amorphous pits, are comparable to the ones observed in Nesher Ramla and to the ones obtained through the experimentation. In five cases the density of the marks is very high, possibly indicating a prolonged use of these items. As in Nesher Ramla, most of the blanks at Quneitra were retouched tools (11 specimens). The emphasis here is mainly on notched pieces (4 specimens), which are also the most common tool type present at the site [39] (Table 3).

The items from Quneitra are considerably shorter than the ones of Nesher Ramla (Table 6 and S1 Data). This difference reflects the general size of artefacts at Quneitra, that are smaller in comparison to other Middle Palaeolithic Levantine sites [47]. Interestingly, while the length of the bulb retouchers of the two assemblages shows great variation, the mean width and thickness are basically identical: 38.1 mm width and 10.5 mm thickness in Quneitra compared to 38.9 mm width and 10.3 mm thickness in Nesher Ramla (Table 6).

The presence of bulb retouchers in another Middle Palaeolithic Levantine site suggests that the employment of flint tools in the manufacture of lithic implements was not a peculiarity of Nesher Ramla’s inhabitants flint-knapping techniques, but, possibly, a more common technological behaviour in the Levant.

## Discussion

The interpretation of bulb retouchers as implements used in the retouching activity is generally accepted [14,19,21,28,31–37]. Another activity that might produce wears on the bulb area of a flint blank is its use as strike-a-lights. However, this do not seem likely, as, previous use-wear studies and experiments showed that macroscopic marks related to fire ignition consist mainly in high-density clusters of incipient cones and rounding of scar ridges and edges rather than the linear pits typical of retouchers [29,53]. In a limited experiment one of us (MO) tried to ignite a spark striking the bulb of percussion of flint flakes against a pyrite lump: the action didn’t produce sparks. It seemed that the smooth surface of the bulb is not suited to create the necessary friction to light a spark. Likewise, use-wear deriving from the strike of flint blanks with the aim of producing sounds consist mainly of high-density clusters of overlapping incipient cones, with surface polish and rare scratches [54,55]. Furthermore, the most diagnostic wear of blanks used as retouchers, the linear pits and streaks, were not attested in the experimental flint blanks used in the production of sounds [54,55]. Therefore, considering the results of our experiment and use-wear study, we accept the general interpretation of bulb retouchers as retouching tools and rule out the hypothesis that the same kind of marks might have originated from their use as strike-a-lights or in the production of sounds.

The results of our experiment show that bulb retouchers are efficient tools for producing a flat, invasive retouch, as the one characterizing the side-scrapers in Nesher Ramla archaeological assemblage (S1 Fig). Based on use-wear analysis, it was possible to sort the bulb retouchers of Nesher Ramla according to the modality in which they were used: oriented parallel or perpendicularly to the edge of the retouched blank. Bulb retouchers seem to have been more commonly used in the parallel mode of retouching in all archaeological units (Table 5), although the perpendicular mode is always present. Our data suggest a relation between metric attributes and retouching mode: smaller, lighter blanks were preferably employed in the perpendicular mode of percussion, while heavier and larger blanks in the parallel mode (Fig 10). Bulb retouchers used in the parallel mode, besides being longer than the ones used in the perpendicular mode, are also significantly longer than most tools at the site. Longer flint artefacts might have offered a better support to the hand of the knapper, allowing a firmer grip while performing the retouch.

### Bulb retouchers in context

The occurrence of bulb retouchers in the archaeological record is not restricted to a specific geographical area (Fig 1). So far, bulb retouchers have been reported in North Africa [32], Caucasus [31], Western Europe [33], Central and Eastern Europe (Jöris, pers comm) [14,19,21,28,34–37] and the Levant. The phenomenon is however constrained in time only to the Middle Palaeolithic, spanning a wide chronological range from at least MIS 9 at Orgnac 3 [1,33] to the most recent ones from Zaskalnaiya cave dated to MIS 3 [14] (S3 Table). Accordingly, they are not associated with any specific cultural complex: they occur in Levantine Mousterian assemblages, Micoquian [14,19,21,28,56], Mousterian [32,36,37,57] and Keilmesser group (Jöris pers comm.) (S3 Table). Bulb retouchers also appear in different contexts (caves, rock-shelters and open-air sites). In Nesher Ramla, Orgnac 3 and several sites of the Crimean Peninsula they are associated with other kinds of retouchers such as bone retouchers [1,14,19,21,28,58] and, in Crimea and Nesher Ramla, pebble retouchers (MP pers obs; S3 Table) [14, 19, 21, 28].

Although Nesher Ramla assemblage is the largest known so far, it is important to notice that bulb retouchers represent only 0.4% of the studied lithic assemblage. While other sites yielded smaller absolute numbers of bulb retouchers, their relevance within the respective assemblages might be greater than in Nesher Ramla. At this stage, it is difficult to quantify the incidence of bulb retouchers in other lithic assemblages, since in many cases the published data are only partial.

### Bulb retouchers blank selection and role as mobile artefacts

Bulb retoucher assemblages from all the different geographic areas share the common characteristic of being composed mainly of retouched pieces, as already noted by Stepanchuk and Sytnyk [37] for the Crimean assemblages. Retouched pieces might have been selected by virtue of their blunted edges, providing a secure grip without injuring the knapper’s hands, as suggested already by Tixier [32]. However, although unretouched blanks were utilized in our experiment, we did not encounter any difficulty related to the sharp edges and none of the knappers suffered injuries. In addition, despite cortical elements being abundant at Nesher Ramla and characterized by blunt edges, they weren’t specifically selected to be used as retouchers.

According to Adler [31], the selection of retouched artefact as bulb retouchers at Ortvale Klde can be related to the greater thickness of these blanks. However, in Nesher Ramla, retouched pieces are not thicker than other blank categories: for instance, primary elements, NBKs and core trimming elements all present thickness value very similar to the ones of retouched pieces. Moreover, in Nesher Ramla, albeit convergent tools represent one of the thinner category of retouched implements, they are frequent in the bulb retouchers assemblage. Conversely, the thicker tools, as notches and denticulates, lightly retouched flakes and blades were seldom employed as retouchers. Hence, thickness, in Nesher Ramla, was not the main criteria affecting the process of selection.

A more likely explanation for the selection of retouched tools is that bulb retouchers were chosen from the general components of the hunter-gatherer’s mobile tool-kits. Retouched tools are usually regarded as the best candidates to be part of the hunter-gatherers mobile tool-kit, as they provide the best ratio of utility to unit of weight [59,60]. Mobile tool-kit components are expected to have a long use-life, to be transportable, general-purpose and flexible tools [51,52, 59–64], expectations often met by retouched implements.

Blanks selected to be used as retouchers at Nesher Ramla and Quneitra possess many of the qualities that characterize mobile tool-kit components. Besides being retouched, they are mostly made from non-local raw materials, in many instances manufactured elsewhere and transported to the site as finished tools. Moreover, several bulb retouchers at Nesher Ramla attest for a complex life-history. The use-wear study conducted on Nesher Ramla bulb retouchers showed that blank used in retouching activities were also utilized in a wide array of other different tasks (Table 7 and Fig 11), although it wasn’t possible to establish if the retouching traces came before or after use-wear generated by other activities. Blanks used as retouchers appear to have been used dynamically, often shifting their technological role in the course of their use-lives, being transformed through retouch or re-sharpened.

The large sample of Nesher Ramla bulb retouchers allowed to highlight a high incidence of intensely retouched convergent tools, a category otherwise underrepresented within the total tool assemblage (Table 3 and S1 Table). This bias might be due to the fact that this class of tools was more often carried around as part of the hunter-gatherer personal gear, serving multiple purposes and used over a prolonged time span. These types appear to be indeed the most mobile artefacts in the tool kit, as they are the tools that more often occur on non-local material (Table 4 and S2 Table), whose sources are located at distances longer than 15 km from the site.

Finally, the option that convergent tools in Nesher Ramla were consciously selected from the pool of tools due to some non-utilitarian properties of these objects needs to be considered. Retouching and the preparation of new tools might have held a symbolic meaning, hence the choice of specific tool types for the manufacturing process. Non-utilitarian behaviours connected to tool manufacturing in the Middle Palaeolithic has already been suggested in light of the discovery of retouchers made on human bones at the site of La Quina [15] and Goyet [13]. Although it is unclear if the knappers were aware that the bone they selected was of human individuals, the fact that they were probably collected and used when the bones were still fresh [13,15], reinforces the hypothesis of a non-utilitarian behaviour [but see 8 for a different view]. In this framework, Nesher Ramla bulb retouchers could have been objects invested with an additional value, prized by their owners and used intensely, over prolonged time, as suggested for the Aurignacian retouchers of La Ferrassie made of carnivore teeth [65].

Unlike in Nesher Ramla, the typology of the bulb retouchers blanks in Quneitra reflects the general composition of the assemblage (Table 3). The small size of other bulb retoucher assemblages, combined with the scarcity of data published for these particular type of tools, does not allow understanding if convergent shaped tools were preferred also in other sites.

### Bulb retouchers and trends of site occupation in Nesher Ramla

In Nesher Ramla, the use of bulb retouchers is a technological trait maintained over a prolonged time span, although in different frequencies. Bulb retouchers characteristics remain extremely homogeneous throughout Nesher Ramla sequence (Unit I-III), in terms of blank selection (retouched blanks, S1 Table), raw material composition (non-local flint, Table 4) and mode of use (parallel or perpendicular, Table 5), suggesting that their role within the tool-kit and the way they were used remained constant through time.

Bulb retouchers are not evenly distributed across Nesher Ramla archaeological sequence: although they represent a small portion of the total lithic assemblage of each archaeological Unit, they occur in higher frequencies within Unit IIB, where they are 2–3 times more abundant than in the other units (Table 2).

One possible explanation for the observed pattern of bulb retouchers variation in Nesher Ramla sequence, is that these changes reflect shifts in settlement patterns adopted by the site inhabitants through time. Bulb retouchers at Nesher Ramla mainly occur on the most curated tool forms (i.e. convergent, *déjeté* side-scrapers and Mousterian points) and their frequencies in the assemblage appear to be strongly correlated to the frequencies of intensely retouched convergent tools (Table 2). The choice to curate a tool-kit is usually regarded as a strategy adopted by highly mobile groups moving in the landscape in order to minimize the risk of being unprepared when an unforeseen need (or opportunity) presents itself [60,61,66–69]. The higher incidence of bulb retouchers and other intensely retouched tools in Unit II (Table 2 and S1 Table) can be connected to a greater curation of the lithic assemblage in this occupational phase, and consequently, to a higher degree of mobility of the site inhabitants. Other archaeological proxies point to a relatively high degree of mobility of Nesher Ramla inhabitants during this phase. Among such proxies are the low values of lithic artefacts density (ca 165 artefacts/m^3^ in Unit IIB and 125 artefacts/m^3^ in Unit IIA), high frequency of retouched tools in the assemblage (S1 Table), the absence of visible spatial features in Unit IIA and their scarce presence in Unit IIB [38,40]. In contrast, the low frequencies of bulb retouchers and intensely retouched tools in Unit III (Table 2), might be related to the more intense human occupations of the site. The latter may be inferred from the high lithic artefacts density (ca 600 artefacts/m^3^), the presence of numerous combustion features, the evidence of hearths rake-out activities [70] and the presence of spatially segregated large accumulations of bones, manuports and lithic artefacts (loci) [38,40,70]. In Unit I, low frequencies of bulb retouchers and intensely retouched tools (Table 3), are associated with a great reliance on local raw material for *débitage* and tool production (Table 4 and S2 Table), small number of retouched implements in the lithic assemblage (Table 2 and S1 Table), and a predominance of simple, lightly-retouched tools within the tool-kit (S1 Table). Unit I exhibit extremely low values of artefacts density (30–60 artefacts/m3), and lack features such as loci and fireplaces. These data, together with geoarchaeological evidences [71], suggest short and sporadic visits to the site, characterized by on-site production and use of most tools, with little input of imported artefacts and a consequent small amount of bulb retouchers within the assemblage.

These conclusions are drawn mainly from data on lithic technology organization at the site, and for the time being should be considered as working hypothesis. The study of the faunal and ground-stone tool assemblages of Nesher Ramla will provide a better understanding of the site function and its changes through time, as well as of the significance of bulb retouchers in relation to past populations mobility.

### Spatial distribution of bulb retouchers in Quneitra

In Quneitra bulb retouchers appear to be spatially segregated to area B, where a wide range of activities took place, especially related to carcass processing, but also flint and basalt knapping [47]. Area A, on the other hand, was possibly a single activity area where only flint knapping took place. Retouched tools appear in both of Quneitra areas, without showing any distinct spatial concentration [47]. The fact that bulb retouchers in Quneitra appear exclusively in area B might be due to the need to frequently re-sharpen tools during intense activities of carcass processing but is also possible that this spatial pattern is a random outcome of the small sample size of Quneitra bulb retoucher assemblage, since they appear in the densest archaeological area.

### Preliminary comparisons with different types of retouchers

As flint blanks, bulb retouchers possess some qualities that made them ideal components of the mobile tool-kit: besides being easily transportable, they are versatile [59,63,64], as they can be used in other activities beside retouch; they are flexible tools [64], in that they can be transformed from one shape to another and re-sharpened several time before discard, thus protracting their use-lives. Moreover, being made on flint, they can function as a “cache” of raw material, as they can change their function and be transformed into cores. The manufacture of a side-scraper, with a continuous row of invasive, scalar retouch, left only few, superficial marks on the bulb retouchers surface. Consequently, the abundant traces on some specimen from the assemblages of Nesher Ramla and of Quneitra, suggest that they could have been used in the production of many tools before being discarded.

These properties of bulb retouchers contrast sharply with the characteristics of bone retouchers. The latter are regarded by many authors as ad-hoc tools, whose blanks were selected from the food waste, attesting to a strictly local raw material provisioning strategy and discarded shortly after their use [16,23,72]. Some estimates based on experimental results suggest that bone retouchers are rapidly exhausted: in most cases they could have been used to manufacture one, or at most few, scraper-like tools, before the worn surface of the tool was no longer efficient in the retouching task [23,72]. Bone retouchers thus, are characterized by a low degree of durability, they do not offer the possibility of being used in other activities beside retouching, and their potential for re-use or recycling is limited. Although light-weight and thus easily transportable, they do not possess other features that usually define mobile tool-kit components, and which would have made them preferable over other retouchers types as mobile elements.

Some authors highlighted a more consistent presence of bone retouchers in sites interpreted as residential camps [9], were hunter-gatherers relied more on a strategy of provisioning of places [60,66,73]. In contrast, high frequencies of bulb retouchers, might suggest a technology more based on curation, in a context of high mobility of the human groups.

In the Levant, the record of retouchers is extremely scarce [74]. Bone retouchers have so far been reported only from the early Upper Palaeolithic layers of Manot Cave [74], the Late Lower Palaeolithic site of Qesem Cave [2,5,75], and, for the Middle Palaeolithic, in Nesher Ramla Unit III and Umm el Tlel [58,76]. Possible pebble retouchers were identified only in Nesher Ramla Unit III (M.P. pers. obs.). The near absence of organic and pebble retouchers in the Middle Palaeolithic of the Levant, despite the impressive record of documented sites may imply a different tradition of retouching techniques in this region during this period. The identification of bulb retouchers in Nesher Ramla and Quneitra partially fills this gap, in addition to the possibility that retouchers were also made on perishable material such as different kinds of woods. Another alternative is that the scarce evidence for retouchers in the Levant is the result of a research bias, as retouchers might have not been recognized in past studies or they might not have been part of past research plans [74]. The ongoing research at Nesher Ramla will possibly contribute in this respect, as most of the faunal and stone assemblages at the site are still under study.

Future studies should involve a comparison of morphometric and mechanical characteristics of different types of retouchers (e.g. bulb, bone and pebble retouchers) in order to better understand the motives which led to the choice of a type instead of another. As bulb retouchers are made on flint, they obviously exhibit very different properties in term of density, elasticity and resistance compared to other type of retouchers and it will be important to evaluate how these qualities might affect the type of retouch obtained. The possibility that different retouchers could have been used in a complementary way during the manufacturing process should also be investigated.

## Conclusions

Bulb retouchers appear over a vast geographic area, spanning from Europe and the Caucasus to the Levant and North Africa, their chronological distribution is however limited, as they only occur within Middle Palaeolithic contexts. Our experiment proved the efficiency of such tools in the manufacture of edges with a flat, regular and invasive retouch, similar to the one characterizing most of Nesher Ramla side-scrapers. The analysis of Nesher Ramla and Quneitra bulb retouchers highlighted a systematic selection of formal tool types as blanks to be used for retouch. Our data suggest that the most likely explanation for this selection is the fact that bulb retouchers were often part of the mobile tool-kit of hunter-gatherers. While our results should be considered as the building blocks of future work hypothesis, they demonstrate that one of the main advantages of bulb retouchers for mobile hunter-gatherers groups resides in their versatility, flexibility and transportability. For the archaeologist, they might be a tale-telling sign of the degree and mode of past mobility.

## Supporting information

S1 Fig Plate of side-scrapers obtained in the experimentation, using the bulb of percussion of flint blanks as retouchers. Plate created with *Artifact3-D* software [1–3], developed by the Computational Archaeology Laboratory, the Institute of Archaeology, the Hebrew University of Jerusalem.
(TIF)

S1 Text List of the attributes used to describe the bulb retouchers active areas. (DOCX)

S2 Text Supplementary information reference list. (DOC)

S1 Table Typological breakdown of bulb retouchers and total tool assemblages by unit in Nesher Ramla. Note that in each unit intensely retouched convergent tools (convergent, *dégetè* side-scrapers and Mousterian points) are always over-represented in the bulb retouchers assemblages compared to the total tool assemblage. The opposite trend can be notet for lightly-retouched tools.
(DOCX)

S2 Table Raw material breakdown by typological classes for each stratigraphic unit at Nesher Ramla. Note the high frequencies of non-local raw material within intensely retouched convergent tools (convergent, *dégetè* side-scrapers and Mousterian points), compared to lightly retouched tools (retouched flakes and blades, retouch on ventral face, *raclettes*).
(DOCX)

S3 Table Summary of the published assemblages of bulb retouchers and their contexts of provenience. (DOCX)

S1 Data Metric and techno- typological attributes of bulb retouchers from Nesher Ramla and Quneitra. (XLSX)
