# Supplementary figures and images for: The bulb retouchers in the Levant: New insights into Middle Palaeolithic retouching techniques and mobile tool-kit composition
Source: PLoS One. 2019 Jul 5;14(7):e0218859. doi: 10.1371/journal.pone.0218859 (PMC6611594; doi:10.1371/journal.pone.0218859)

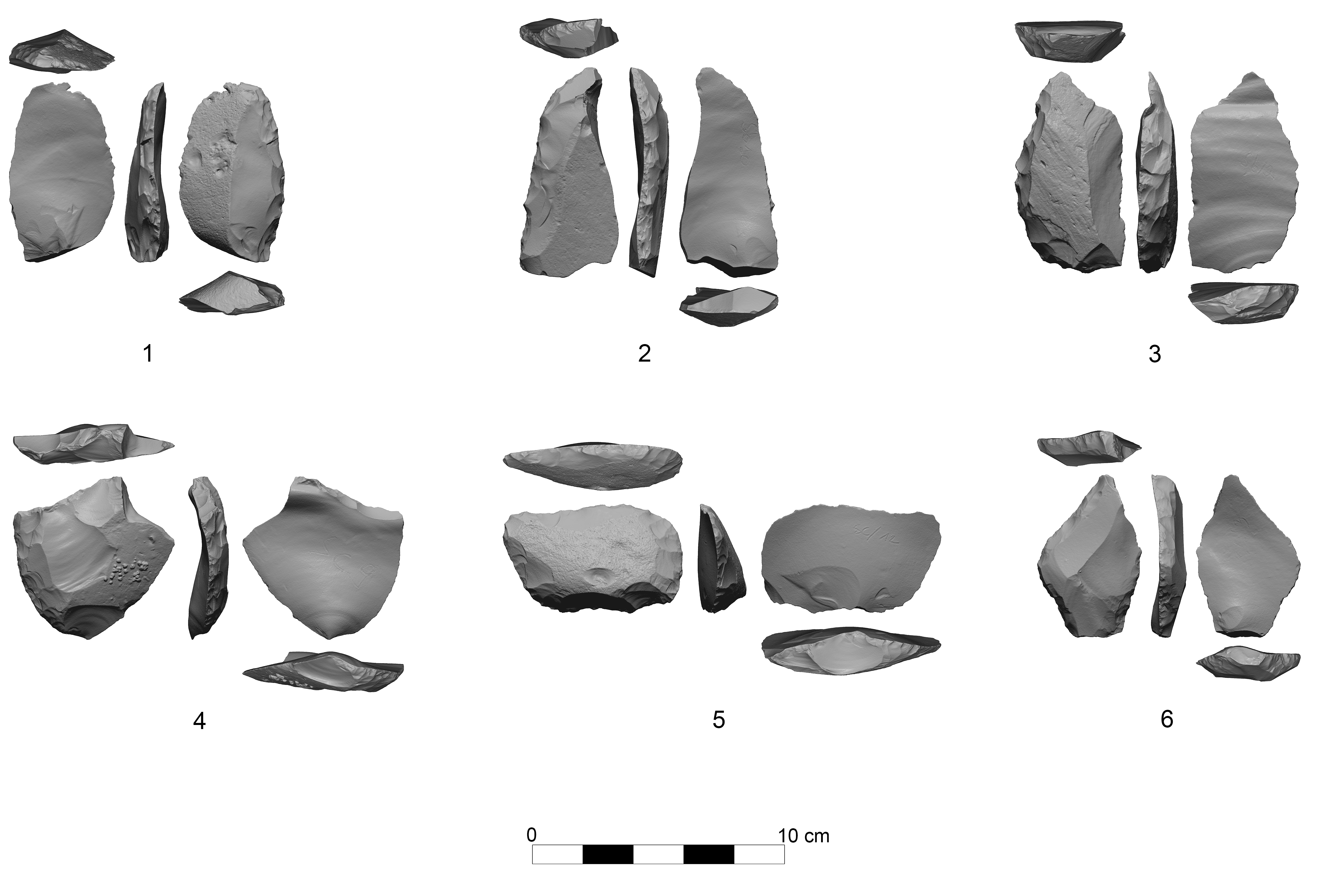

Supplement: S1 Fig — Plate created with Artifact3-D software [1–3], developed by the Computational Archaeology Laboratory, the Institute of Archaeology, the Hebrew University of Jerusalem. (TIF) [file pone.0218859.s001.tif]
