# Supplementary material for: The bulb retouchers in the Levant: New insights into Middle Palaeolithic retouching techniques and mobile tool-kit composition
Source: PLoS One. 2019 Jul 5;14(7):e0218859. doi: 10.1371/journal.pone.0218859 (PMC6611594; doi:10.1371/journal.pone.0218859)
Supplement: S1 Text — (DOCX) [file pone.0218859.s002.docx]

# Bulb retouchers active area attribute list

Macroscopic marks position

- Bulb central

-Bulb distal

- Bulb left

- Bulb right

- Dorsal

- On the ventral face

- On fracture

Shape of the marks area

- Linear

- Cluster

- Sparse

Marks density

- High

- Medium

- Low

Diameter of the scar field (lenght mm)

Diameter of the scar field (width mm)

Minimum distance of marks from the striking platform (mm)

Macroscopic marks type

- Pits

- Incipient cones

- Pits and incipient cones

Number of active areas

Orientation of striations compared to the tool long axis

- Perpedicular

- Perpendicular and parallel

- Perpendicular and oblique

- Parallel

- Parallel and oblique

- Oblique

Location of the striations

- On bulb central

- Left slope of the bulb

- Right slope of the bulb

Use as bulb retoucher last stage of use?

- No, the tool is too small

- No, scars cut into the marks area

- Indeterminate
