# Supplementary material for: The bulb retouchers in the Levant: New insights into Middle Palaeolithic retouching techniques and mobile tool-kit composition
Source: PLoS One. 2019 Jul 5;14(7):e0218859. doi: 10.1371/journal.pone.0218859 (PMC6611594; doi:10.1371/journal.pone.0218859)
Supplement: S2 Text — (DOC) [file pone.0218859.s003.doc]

**Supplementary information references**

1. Grosman L. Reaching the Point of No Return: The Computational Revolution in Archaeology. Annu Rev Anthropol. 2016; 45:129–145. doi: 10.1146/annurev-anthro-102215-095946
2. Grosman L, Karasik A, Harush O, Smilansky U. Archaeology in Three Dimensions: Computer-Based Methods in Archaeological Research. J East Mediterr Archaeol Herit Stud. 2014; 2:48–64. doi: 10.5325/jeasmedarcherstu.2.1.0048
3. Grosman L, Smikt O, Smilansky U. On the application of 3-D scanning technology for the documentation and typology of lithic artifacts. J Archaeol Sci. 2008; 35:3101–3110. doi: 10.1016/j.jas.2008.06.011
4. Zaidner Y, Frumkin A, Porat N, Tsatskin A, Yeshurun R, Weissbrod L. A series of Mousterian occupations in a new type of site: The Nesher Ramla karst depression, Israel. J Hum Evol. 2014;66(1):1–17.
5. Goren-Inbar N. Quneitra: a Mousterian site on the Golan Heights. Jerusalem: Institute of Archaeology: The Hebrew University of Jerusalem; 1990.
6. Adler DS. Late Middle Palaeolithic Patterns of Lithic Reduction, Mobility, and Land Use in the Southern Caucasus. PhD Thesis, Harvard University Cambridge, Massachusetts; 2002.
7. Adler DS, Tushabramishvili N. Middle Palaeolithic patterns of settlement and subsistence in the southern Caucasus. In: Conard NJ, editor. Settlement Dynamics of the Middle Palaeolithic and Middle Stone Age II. Tübingen: Kerns Verlag; 2004. p. 91–132.
8. Praslov ND. Rannii Paleolit Severo-Vostochnogo Priazov’ya i Nizhnego Dona. (The Early Paleolithic of the North-Eastern Azov Valley and Low Don). Leningrad: Nauka; 1968. p. 155.
9. Cohen VY, Stepanchuk VN. Late Middle and Early Upper Paleolithic evidence from the East European Plain and Caucasus: A new look at variability, interactions, and transitions. J World Prehistory. 1999;13(3):265–319.
10. Kolosov YUG, Stepanchuk V. New type of Middle Palaeolithic Industry in Eastern Crimea. Arceologické Rozhl. 1997;XLIX:124–45.
11. Stepanchuk VN. Kiik-Kobian: A Distinct Mousterian Industry in the Crimea. Archeol Rozhl. 1992;XLIV(4):505–23.
12. Stepanchuk VN, Vasilyev S V., Khaldeeva NI, Kharlamova N V., Borutskaya SB. The last Neanderthals of Eastern Europe: Micoquian layers IIIa and III of the site of Zaskalnaya VI (Kolosovskaya), anthropological records and context. Quat Int. 2017;428:132–50.
13. Plisson H. Technologie et tracéologie des outils lithiques moustériens en Union Soviétique: Les travaux de V. E. Shchelinskii. In Otte M, editor. L’Homme de Neandertal. Vol. 4: La Technique. Liège: Études et Recherce Archéologiques de L’Université de Liège; 1988;1:121–68.
14. Stepanchuk VN, Sytnyk O. The Chaînes Opératoires of Levallois site Pronyatyn, Western Ukraine. Prehistoire Eur. 1999;13:33–67.
15. Chabai VP. Chokurcha I Unit IV: Artifacts. In: Chabai VP, Monigal K, Marks AE, editors. The Middle Paleolithic and Early Upper Paleolithic of Eastern Crimea. Liège: ERAUL; 2004. p. 377–416.
16. Mathias C, Viallet C. On the possible use of flake-bulbs for retouch during the early Middle Palaeolithic in southeastern France: First results of an experimental approach. BUTLLETÍ Arqueol. 2018;40:323–8.
17. Moigne AM, Valensi P, Auguste P, García-Solano J, Tuffreau A, Lamotte A, et al. Bone retouchers from Lower Palaeolithic sites: Terra Amata, Orgnac 3, Cagny-l’Epinette and Cueva del Angel. Quat Int. 2016;409:195–212.
18. Jöris O. Bifacially backed knives (Keilmesser) in the central European Middle Palaeolithic. In: Goren-Inbar N, Sharon G, editors. Axe age: Acheulian tool-making from quarry to discard. London: Equinox; 2006. P. 287-310.
19. Tixier J. Outils Moustériens a bulbe “piqueté” (Retaimia, Algérie). In: Otte M, editor. À la recherche de L’Homme préhistorique. ERAUL 95. Liège; 2000. p. 125–30.
