# Supplementary material for: The bulb retouchers in the Levant: New insights into Middle Palaeolithic retouching techniques and mobile tool-kit composition
Source: PLoS One. 2019 Jul 5;14(7):e0218859. doi: 10.1371/journal.pone.0218859 (PMC6611594; doi:10.1371/journal.pone.0218859)
Supplement: S1 Table — Note that in each unit intensely retouched convergent tools (convergent, dégetè side-scrapers and Mousterian points) are always over-represented in the bulb retouchers assemblages compared to the total tool assemblage. The opposite trend can be notet for lightly-retouched tools. (DOCX) [file pone.0218859.s004.docx]

| **Typology** | **Unit III bulb retouchers** | | **Unit III total tool assemblage** | | **Unit IIB bulb retouchers** | | **Unit IIB total tool assemblage** | | **Unit IIA bulb retouchers** | | **Unit IIA total tool assemblage** | | **Unit I bulb retouchers** | | **Unit I total tool assemblage** | |
| --- | --- | --- | --- | --- | --- | --- | --- | --- | --- | --- | --- | --- | --- | --- | --- | --- |
|  | n | % | n | % | n | % | n | % | n | % | n | % | n | % | n | % |
| Simple side-scrapers | 7 | 50.0 | 535 | 43.6 | 50 | 46.3 | 1,000 | 40.3 | 3 | 23.1 | 62 | 22.5 | 3 | 37.5 | 89 | 28.3 |
| Double side-scrapers | 1 | 7.1 | 50 | 4.1 | 7 | 6.5 | 140 | 5.6 | - | - | 12 | 4.3 | - | - | 14 | 4.4 |
| Convergent side-scrapers | 1 | 7.1 | 20 | 1.6 | 10 | 9.3 | 97 | 3.9 | 3 | 23.1 | 11 | 4.0 | - | - | 7 | 2.2 |
| *Déjeté* side-scrapers | 1 | 7.1 | 13 | 1.1 | 5 | 4.6 | 75 | 3.0 | - | - | 3 | 1.1 | - | - | 2 | 0.6 |
| Transversal side-scrapers | - | - | 20 | 1.6 | 1 | 0.9 | 61 | 2.5 | - | - | 2 | 0.7 | - | - | 6 | 1.9 |
| Other side-scrapers | 2 | 14.3 | 7 | 0.6 | 2 | 1.9 | 15 | 0.6 | - | - | 3 | 1.1 | - | - | 4 | 1.3 |
| Mousterian points | - | - | 12 | 1.0 | 24 | 22.2 | 120 | 4.8 | 4 | 30.8 | 14 | 5.1 | 1 | 12.5 | 10 | 3.2 |
| Retouched Levallos points | - | - | 20 | 1.6 | - | - | 29 | 1.2 | 1 | 7.7 | 12 | 4.3 | 2 | 25.0 | 8 | 2.5 |
| Retouched flakes and blades | - | - | 224 | 18.3 | 4 | 3.7 | 340 | 13.7 | - | - | 36 | 13.0 | 1 | 12.5 | 59 | 18.7 |
| Notches and denticulates | - | - | 38 | 3.1 | 2 | 1.9 | 85 | 3.4 | - | - | 26 | 9.4 | - | - | 15 | 4.8 |
| Other tools | 1 | 7.1 | 204 | 16.6 | 1 | 0.9 | 215 | 8.7 | - | - | 34 | 12.3 | - | - | 36 | 11.4 |
| Broken tools | 1 | 7.1 | 83 | 6.8 | 2 | 1.9 | 307 | 12.4 | 2 | 15.4 | 61 | 22.1 | 1 | 12.5 | 65 | 20.6 |
| Tot Retouched pieces | 14 | 82.4 | 1,226 | 11.0 | 108 | 90.0 | 2,485 | 12.0 | 13 | 92.9 | 276 | 6.8 | 8 | 100.0 | 315 | 7.7 |
| Un-retouched NBKs | 1 | 5.9 | 886 | 7.9 | 5 | 4.2 | 1,406 | 6.8 | - | - | 325 | 8.0 | - | - | 292 | 7.1 |
| Other un-retouched blanks | 2 | 11.8 | 9,048 | 81.1 | 7 | 5.8 | 16,747 | 81.0 | 1 | 7.1 | 3,466 | 85.2 | - | - | 3,478 | 85.1 |
| **TOTALS** | **17** | 100.0 | **11,160** | 100.0 | **120** | 100.0 | **20,638** | 100.0 | **14** | 100.0 | **4,067** | 100.0 | **8** | 100.0 | **4,085** | 100.0 |
