# Supplementary material for: The bulb retouchers in the Levant: New insights into Middle Palaeolithic retouching techniques and mobile tool-kit composition
Source: PLoS One. 2019 Jul 5;14(7):e0218859. doi: 10.1371/journal.pone.0218859 (PMC6611594; doi:10.1371/journal.pone.0218859)
Supplement: S2 Table — Note the high frequencies of non-local raw material within intensely retouched convergent tools (convergent, dégetè side-scrapers and Mousterian points), compared to lightly retouched tools (retouched flakes and blades, retouch on ventral face, raclettes). (DOCX) [file pone.0218859.s005.docx]

|  | **Unit I** | | | | | | **Unit IIA** | | | | | | **Unit IIB** | | | | | | **Unit III** | | | | | |
| --- | --- | --- | --- | --- | --- | --- | --- | --- | --- | --- | --- | --- | --- | --- | --- | --- | --- | --- | --- | --- | --- | --- | --- | --- |
|  | **Mishash** | | **Non-Mishash** | | **Tot** | | **Mishash** | | **Non-Mishash** | | **Tot** | | **Mishash** | | **Non-Mishash** | | **Tot** | | **Mishash** | | **Non-Mishash** | | **Tot** | |
|  | n | % | n | % | n | % | n | % | n | % | n | % | n | % | n | % | n | % | n | % | n | % | n | % |
| **Simple side-scrapers** | 40 | 75 | 13 | 25 | 53 | 100 | 12 | 55 | 10 | 45 | 22 | 100 | 349 | 61 | 220 | 39 | 569 | 100 | 334 | 63 | 197 | 37 | 531 | 100 |
| **Double side-scrapers** | 4 | 44 | 5 | 56 | 9 | 100 | 6 | 75 | 2 | 25 | 8 | 100 | 43 | 49 | 45 | 51 | 88 | 100 | 20 | 40 | 30 | 60 | 50 | 100 |
| **Convergent side-scrapers** | 4 | 80 | 1 | 20 | 5 | 100 | 3 | 50 | 3 | 50 | 6 | 100 | 24 | 45 | 29 | 55 | 53 | 100 | 7 | 35 | 13 | 65 | 20 | 100 |
| ***Déjeté* side-scrapers** | - | - | 3 | 100 | 3 | 100 | 2 | 67 | 1 | 33 | 3 | 100 | 30 | 53 | 27 | 47 | 57 | 100 | 5 | 38 | 8 | 62 | 13 | 100 |
| **Transversal side-scrapers** | 6 | 100 | - | - | 6 | 100 | 1 | 50 | 1 | 50 | 2 | 100 | 18 | 53 | 15 | 47 | 33 | 100 | 11 | 55 | 9 | 45 | 20 | 100 |
| **Other side-scrapers** | 3 | 100 | - | - | 3 | 100 | - | - | - | - | - | - | 7 | 78 | 2 | 22 | 9 | 100 | - | - | - | - | - | - |
| **Mousterian points** | 4 | 67 | 2 | 33 | 6 | 100 | 3 | 43 | 4 | 57 | 7 | 100 | 31 | 37 | 52 | 63 | 83 | 100 | 3 | 25 | 9 | 75 | 12 | 100 |
| **Retouched Levallois points** | 3 | 50 | 3 | 50 | 6 | 100 | 6 | 67 | 3 | 33 | 9 | 100 | 11 | 58 | 8 | 42 | 19 | 100 | 16 | 80 | 4 | 20 | 20 | 100 |
| **Retouched flakes and blades** | 22 | 67 | 11 | 33 | 33 | 100 | 10 | 56 | 8 | 44 | 18 | 100 | 164 | 75 | 54 | 25 | 218 | 100 | 154 | 68 | 72 | 32 | 226 | 100 |
| **Retouch on ventral face** | 2 | 67 | 1 | 33 | 3 | 100 | 1 | 100 | - | - | 1 | 100 | 23 | 88 | 3 | 12 | 26 | 100 | 7 | 100 | - | - | 7 | 100 |
| **Notches and denticulates** | 7 | 88 | 1 | 12 | 8 | 100 | 7 | 88 | 1 | 12 | 8 | 100 | 31 | 74 | 11 | 26 | 42 | 100 | 30 | 79 | 8 | 21 | 38 | 100 |
| ***Raclette*** | 2 | 67 | 1 | 33 | 3 | 100 | - | - | - | - | - | - | 39 | 78 | 11 | 22 | 50 | 100 | 45 | 78 | 13 | 22 | 58 | 100 |
| **Resharpened pieces** | 9 | 82 | 2 | 18 | 11 | 100 | 6 | 75 | 2 | 25 | 8 | 100 | 35 | 70 | 15 | 30 | 50 | 100 | 81 | 66 | 41 | 34 | 122 | 100 |
| **Other tools** | 2 | 100 | - | - | 2 | 100 | 3 | 100 | - | - | 3 | 100 | 13 | 76 | 4 | 24 | 17 | 100 | 17 | 85 | 3 | 25 | 20 | 100 |
| **Tot retouched pieces** | **108** | **72** | **43** | **28** | **151** | **100** | **60** | **63** | **35** | **37** | **95** | **100** | **818** | **62** | **496** | **38** | **1,314** | **100** | **730** | **64** | **407** | **36** | **1,137** | **100** |
| **Intensely retouched convergent tools** | 8 | 57 | 6 | 43 | 14 | 100 | 8 | 50 | 8 | 50 | 16 | 100 | 85 | 44 | 108 | 56 | 193 | 100 | 15 | 33 | 30 | 67 | 45 | 100 |
| **Lightly retouched tools** | 26 | 67 | 13 | 33 | 39 | 100 | 11 | 58 | 8 | 42 | 19 | 100 | 226 | 77 | 68 | 23 | 294 | 100 | 206 | 71 | 85 | 29 | 569 | 100 |
