# Supplementary material for: The bulb retouchers in the Levant: New insights into Middle Palaeolithic retouching techniques and mobile tool-kit composition
Source: PLoS One. 2019 Jul 5;14(7):e0218859. doi: 10.1371/journal.pone.0218859 (PMC6611594; doi:10.1371/journal.pone.0218859)
Supplement: S3 Table — (DOCX) [file pone.0218859.s006.docx]

| Site | Layer | Location | Culture | Dates BP | Type of site | N of bulb retouchers | Bone retouchers | Pebble retouchers |
| --- | --- | --- | --- | --- | --- | --- | --- | --- |
| Nesher Ramla ^[4]^ | I | Central Israel | Levantine Mousterian | 130-80 ka | Open-air | 8 | ? | ? |
|  | IIA |  | Levantine Mousterian |  |  | 14 | ? | ? |
|  | IIB |  | Levantine Mousterian |  |  | 120 | ? | ? |
|  | III |  | Levantine Mousterian |  |  | 17 | yes | yes |
| Quneitra ^[5]^ | 3 | Golan Heights | Levantine Mousterian | 54 Ka | Open-air | 13 | no | no |
| Ortvale Klde ^[6-7]^ | 6 | Georgia | Mousterian | 60-35 ka | Rockshelter | 3 | no | no |
|  | 7 |  | Mousterian |  |  | 1 | no | no |
|  | 9 |  | Mousterian |  |  | 3 | no | no |
|  | 10 |  | Mousterian |  |  | 3 | no | no |
| Rojok I ^[8-9]^ | 2-4 | Crimea | Micoquian |  |  | 2? | ? | ? |
| Alyoshin Grot ^[10]^ | 2 | Crimea | Typical Mousterian |  | Rockshelter | 5 | no | no |
| Prolom I ^[11]^ | lower layer | Crimea | Micoquian |  | Cave | 1 | yes | yes |
|  | upper layer |  | Micoquian |  |  | several | yes | yes |
| Kiik-Koba ^[11]^ | upper layer | Crimea | Micoquian |  | Cave | 1 | yes | yes |
| Zaskalnaya VI ^[12]^ | III | Crimea | Micoquian | 35-40 ka | Cave | 36 | yes | yes |
|  | IIIa |  | Micoquian |  |  | 15 | yes | yes |
| GABO ^[13]^ |  | Crimea | Micoquian |  | Cave | 2 | ? | ? |
| Chokurcha I ^[14]^ | IV-I | Crimea | Micoquian | > 45 ka | Rockshelter | 1 | yes | yes |
| P Pronyatyn ^[15]^ | ? | Ukraine | Typical Mousterian |  | Open-air | 12 | ? | no |
| Orgnac 3 ^[16-17]^ | 6 | France | Mousterian | MIS 9 | Cave | 1 | yes | ? |
| Balver Höhle ^[18; Jöris pers comm]^ |  | Germany | Keilmesser group | Late MP | Cave | few | ? | ? |
| Retaimia ^[19]^ | ? | Algeria | Mousterian |  | Cave | 3 | ? | ? |
